# Unlocking the financing potential of forest-based carbon assets: a valuation framework for pledge lending under uncertainty in China

**DOI:** 10.1038/s41598-026-53268-y

**Published:** 2026-05-18

**Authors:** Yan ZHANG, Jingyu ZHANG

**Affiliations:** 1 Department School of Economics, Yunnan University, Kunming, China; 2 Centre of Development Studies, University of Cambridge, Cambridge, UK

**Keywords:** Forest-based carbon assets, Pledge lending, Carbon asset valuation, Real options and forecasting modelling, Sustainable finance innovation, Climate sciences, Ecology, Ecology, Environmental sciences, Environmental social sciences

## Abstract

Forest-based carbon assets are increasingly proposed as collateral for green credit, yet a central problem remains unresolved: a higher collateral valuation does not necessarily translate into greater lendable value. Here we develop a contract-consistent framework for pricing forest-based carbon collateral under uncertainty and apply it to the Ning’er afforestation case in Yunnan, China, covering 51,365 mu and 719,680 t ${CO}_{2}$ e of expected carbon assets. The framework combines weekly carbon-price forecasting from thin and irregular China Certified Emission Reduction (CCER) trading data with threshold-based option valuation and a lender-oriented pledge-rate equation anchored to the observed three-year loan structure. Using weekly pre-loan transactions from 31 August 2018 to 30 September 2022, we show that incorporating uncertainty and timing flexibility raises the option-adjusted collateral value to CNY 71.87 million. However, once effective deliverability, downside protection and prudential threshold calibration are imposed, the model-implied lower-bound pledgeable amount is only CNY 5.92 million, below the realised loan of CNY 12 million. Sensitivity analysis further shows that financing capacity is jointly shaped by carbon price, effective deliverability, volatility identification and threshold calibration. Our results show that valuing forest-based carbon assets for lending is not a single-stage asset-pricing problem, but a constrained translation problem between economic collateral value and lender-recognised lendable value.

**Supplementary Information:**

The online version contains supplementary material available at 10.1038/s41598-026-53268-y.

## Introduction

Addressing climate change has become a central pillar of global sustainable development^1,2^. Since the adoption of the United Nations Framework Convention on Climate Change (UNFCCC) in 1992, a series of legally binding international agreements—such as the Kyoto Protocol and the Paris Agreement—have catalysed the development of carbon markets and formalised national emission reduction commitments^3–5^. In alignment with this trajectory, China has undertaken extensive efforts to strengthen its climate governance framework, including the establishment of both voluntary and compliance-based carbon trading mechanisms^6^. The launch of the national emissions trading scheme (ETS) in July 2021 marked a significant step towards institutionalising carbon pricing across sectors^7^. Voluntary carbon markets, however, have suffered a crisis of confidence, fuelled not only by inconsistent standards and policy uncertainty, but also by growing evidence that some forest-related offsets may substantially overstate claimed mitigation effects when baseline methodologies are weakly grounded^1,2,8–11^. Recent work also shows that permanence cannot be taken for granted even where forest offsets adopt formal insurance mechanisms: current buffer-pool contributions may be materially insufficient to cover disturbance-driven carbon losses, with required buffer sizes varying systematically by forest age and disturbance regime^12^. Forests and other nature-based options absorb around 7.6 Gt CO₂ each year, support roughly 1.6 billion people and could deliver over one-third of the mitigation required by 2030, but currently attract less than 3% of climate finance; when credibly measured and verified, forest credits command price premia^13,14^. Although near-term volatility and a projected shortfall of high-quality removals of about 60 Mt per year by 2030 are likely, tailwinds are strengthening via Article 6.4 of the Paris Agreement, growing CORSIA demand (approximately 135–180 Mt by 2026) and IC-VCM’s Core Carbon Principles^15,16^. Given forests store an estimated 861 Gt of carbon and could store a further 226 Gt, credible forest credits remain essential, with offsets confined to residual emissions and governed by robust, transparent regulation^17–19^.

Within this policy context, forest-based carbon sinks have emerged as a strategic component of China’s dual carbon goals: peaking carbon emissions by 2030 and achieving carbon neutrality by 2060^13,20,21^. Through afforestation and reforestation programmes, China has significantly expanded its forest carbon stock, with planted forests playing an increasingly important role in national carbon sequestration efforts^14^. As a result, forest-based carbon assets are now transitioning from ecological resources to monetisable, financeable assets within the broader climate finance system^22–24^.

Nevertheless, the integration of forest carbon into formal financial systems remains constrained by long project cycles, delayed cash flows, and valuation uncertainties^25^. Recent evidence further suggests that durability risk may be substantially underinsured in existing forest offset designs, implying that nominal carbon claims should not be treated as fully financeable collateral quantities without additional prudential adjustment^12^. Although initial explorations of pledge-based lending have emerged, using forest carbon credits or expected carbon yields as collateral, progress has remained limited owing to the absence of reliable pricing models, standardised risk-assessment frameworks, and credible asset classification systems^26^. Existing valuation methods, often based on discounted cash flow models, fall short in capturing the option value and market volatility inherent to carbon assets, thereby impeding effective financial engagement by lenders.

While scholarly work on carbon markets and emissions trading has expanded considerably, far less attention has been devoted to the pricing, risk treatment, and collateralisation of forest-based carbon assets within pledge-based financing arrangements. In particular, a theoretical and empirical gap remains in integrating option-based valuation with data-driven forecasting to recover the latent economic value of such assets under carbon-price uncertainty^27^. Carbon-related value claims are methodologically contingent, so financing cannot treat nominal carbon quantity as automatically bankable^11^. To address this gap, this study develops a contract-consistent pricing framework for forest-based carbon pledge lending that distinguishes explicitly between two analytically different objects: the economic value of carbon collateral and its lender-relevant lendable value. The framework combines three linked components. First, it uses a weekly CNN–LSTM forecasting module to recover a forward-looking market environment under thin and irregular CCER trading conditions. Second, it applies option-based valuation in which the strike is defined not as a forecasted future price, but as a contract-grounded debt-coverage threshold. Third, it derives a lender-oriented pledge rate through a break-even condition that maps option-adjusted collateral value into a model-implied pledgeable amount under downside risk. The contribution of the study therefore does not lie in showing that more sophisticated valuation automatically justifies more generous lending. Rather, it lies in demonstrating why natural-capital collateral can be revalued upward while still yielding a materially lower lendable amount once implementation risk, threshold calibration and prudential lending logic are taken seriously. In this sense, the paper contributes not only a new empirical application, but also a more general climate-finance insight: valuing carbon-based natural capital for lending is a two-stage problem of asset pricing and prudential translation, and the two stages need not move in the same direction.

### Pledge financing for forest-based carbon assets

Carbon finance has gained increasing academic attention as a pivotal mechanism for reallocating resources and supporting the low-carbon transition^28,29^. While carbon pricing lays the groundwork for market incentives, many scholars argue that its effectiveness depends significantly on the availability and design of accompanying financial instruments^30^. Some scholars emphasised the limited role of pricing alone and underscored the importance of credit flows in guiding firms’ investment decisions^31,32^, similarly, drawing on endogenous growth theory, highlighted the positive externalities of carbon finance in accelerating innovation in clean technologies^33^ These insights collectively suggest that the institutional value of carbon finance lies not only in price signalling, but also in its leverage as a financing intermediary^34^.

In the Chinese context, forest-based carbon sinks have emerged as a major vehicle for achieving national carbon neutrality targets^35^. With rapid expansion in afforestation efforts and improvements in forest carbon accounting systems, forest carbon projects are increasingly recognised as potential financial assets^36^. Existing literature has categorised a range of forest carbon finance instruments, including pledge loans, forward purchase agreements, and carbon insurance, all offering a preliminary product taxonomy for the sector^37–39^. While pledge financing has shown promise in addressing the structural mismatch between the long payback periods of forest carbon projects and their short-term financing needs, practical implementation remains constrained by valuation ambiguity, credit risk, and limited institutional participation.

Scholars have proposed various approaches to valuing forest carbon assets, including cost-based, income-based, market-based, and shadow pricing methods^40,41^. However, these approaches are subject to significant limitations. Cost-based models often underestimate asset value by ignoring future revenue potential; income-based methods inadequately capture uncertainty and policy risk; and market-based approaches also face major practical constraints in China’s nascent carbon market, including low liquidity, fragmented regulatory arrangements, entitlement ambiguity, and complex registration and verification procedures for forestry offsets^42^. Although some researchers have introduced real options and shadow pricing techniques to address these gaps, these methods often face challenges in parameter estimation, model complexity, and practical applicability.

In sum, the current literature on pledge financing for forest-based carbon assets suffers from two core limitations: a lack of dynamic valuation models that reflect price volatility and uncertainty, and insufficient integration of predictive mechanisms to estimate future asset value. Without a scientifically grounded and operationally feasible pricing framework, it is difficult to unlock the full financing potential of forest carbon assets or to scale up their role within green financial systems. This study responds to this gap by proposing a dynamic valuation model that integrates real options theory with carbon price forecasting to enhance the financial viability and credibility of forest-based carbon asset lending.

### Valuation of forest-based carbon assets through the real options lens

Real options theory, initially proposed by Stewart Myers in 1977, extends the principles of financial options into real asset evaluation. It moves beyond static discounted cash flow models by treating investment decisions under uncertainty as embedded options, thereby allowing for the quantification of managerial flexibility and strategic timing. By capturing the value of delaying, expanding, or abandoning a project in response to external volatility, the real options framework is particularly suited to long-term projects characterised by price fluctuations, irreversible costs, and policy sensitivity.

Forest-based carbon asset projects typify such conditions. Their economic returns are highly sensitive to carbon price trajectories, regulatory dynamics, and biological growth cycles^13,14^. These characteristics confer upon them an inherent “optionality” that conventional valuation approaches struggle to account for. In response, a growing body of research has applied real options analysis (ROA) to forest carbon projects, aiming to capture their latent value under volatile and evolving market conditions. For example, evidence from the New Zealand Emissions Trading Scheme shows that, when carbon and timber prices are stochastic, real-options valuation can materially increase bare-land value, lengthen optimal rotation ages, and generate decision-relevant harvest thresholds governing whether to harvest or to wait. This literature highlights that forestry carbon value depends not only on expected revenues, but also on the timing flexibility embedded in policy-linked price uncertainty^43^.

The methodological evolution within this field has proceeded along two primary lines. On one hand, formal option pricing models—such as Black–Scholes and binomial tree structures—have been adapted to quantify the contingent value of forest carbon under various market states. On the other hand, decision-oriented models have sought to embed project flexibility directly into carbon investment and financing strategies, incorporating parameters such as deferred credit issuance, price triggers, and policy windows. These approaches enhance the valuation toolkit by recognising the interplay between asset volatility and managerial discretion, offering more realistic assessments of investment risk and timing.

Nonetheless, existing applications of real options theory to forest-based carbon valuation face two major constraints. First, the accuracy of model outputs remains highly sensitive to the pre-determined values of key parameters, such as carbon price volatility and risk-free rates, which are often assumed rather than empirically derived. Second, standard real options models lack the capacity to capture nonlinear dynamics inherent in emerging carbon markets, including abrupt policy shifts and behavioural feedbacks. These limitations call for an integrated approach—one that combines the theoretical robustness of real options analysis with data-driven forecasting techniques—to enhance responsiveness to market uncertainty and support more adaptive and bankable valuation frameworks for forest carbon assets.

### Advances in carbon price forecasting: from statistical models to deep learning approaches

Carbon pricing plays a pivotal role in the valuation of forest-based carbon assets^44^. As a key determinant of economic returns and project risk, its fluctuations directly affect the embedded option value of carbon projects and their viability as collateralised financial instruments. Consequently, accurately forecasting carbon price dynamics has become a central methodological challenge in carbon finance research^45^. Existing studies have broadly followed two methodological trajectories: traditional econometric models rooted in statistical theory, and machine learning-based approaches that embrace non-linear, data-driven modelling paradigms^40^.

Early work predominantly employed time-series techniques such as GARCH, ARMA, and linear regression models, often enhanced with decomposition or transformation techniques (e.g., empirical mode decomposition, fractal Brownian motion) to improve model responsiveness to short-term price volatility. These models benefit from theoretical tractability and parameter interpretability, making them suitable for short-horizon forecasting and volatility analysis. However, their performance is constrained by the need to impose distributional assumptions, and they often fail to capture the complex, non-linear, and non-stationary patterns evident in real-world carbon price series—particularly over longer timeframes.

In response to the growing complexity of carbon markets, recent research has shifted towards machine learning (ML) and deep learning (DL) approaches. These non-parametric methods are better suited to model structural complexity and time-dependent patterns without requiring prior assumptions about the data-generating process. Techniques such as random forests, BP neural networks, and recurrent neural networks (e.g. LSTM) have demonstrated notable gains in predictive accuracy, particularly when working with high-dimensional, multi-source datasets. Hybrid deep learning models—such as CNN-LSTM and Transformer-LSTM architectures—have further enhanced prediction performance by combining spatial feature extraction with temporal sequence learning. These models excel at capturing periodic fluctuations, regime shifts, and exogenous shocks in carbon markets.

Empirical findings indicate that deep learning models significantly outperform traditional methods in forecasting carbon price movements under conditions of high volatility and structural uncertainty^46–48^. Consequently, deep learning has largely supplanted shallow neural networks as the dominant paradigm in financial time-series forecasting. The overall trend suggests a paradigmatic shift from linear, assumption-driven modelling towards non-linear, adaptive learning-based systems capable of extracting latent structures from complex datasets.

Nonetheless, challenges remain. First, the ‘black-box’ nature of deep learning models limits their transparency and interpretability, which are critical for policy evaluation and financial valuation contexts. Second, while these models provide superior predictive accuracy, they are often difficult to integrate directly into valuation frameworks based on risk–return logic, such as real options models or carbon asset pricing structures. Thus, a key methodological challenge lies in balancing predictive power with model interpretability, and in designing architectures that are both accurate and operationally compatible with carbon finance applications.

### Pledge rate modelling

The pledge rate—the haircut applied to collateral in secured lending—is a critical determinant of both borrower financing costs and lender risk exposure. Three main strands of methodology have been developed to estimate appropriate pledge rates, each resting on different theoretical foundations and facing its own challenges.

First, option-pricing approaches treat the pledged asset as the underlying of an embedded option, using Black–Scholes or its extensions to link the haircut dynamically to parameters such as volatility, time to maturity and risk premia. Under this framework, higher collateral volatility leads to deeper haircuts (lower pledge rates), while higher required returns allow shallower haircuts. However, these models assume continuous trading and log-normal price processes, and they require reliable estimates of volatility and interest rates—conditions that are often unattainable in illiquid or opaque markets.

Second, risk-assessment frameworks derive pledge rates by constraining expected losses under extreme but plausible scenarios. By estimating value-at-risk or conditional value-at-risk—often incorporating liquidity adjustments, correlation structures and tail‐risk corrections—these models can be calibrated to diverse asset classes, from metals to equities. Their flexibility, however, depends heavily on the quality of historical data and on distributional assumptions, and they must be recalibrated frequently to retain accuracy as market conditions evolve.

Third, default-probability methods infer pledge rates from the borrower’s likelihood of default and the lender’s recovery assumptions. These models map estimated default probabilities onto expected loss‐given‐default, thereby tying the haircut directly to credit risk. Although intuitively appealing and well aligned with standard credit-risk management, the approach demands robust credit-scoring infrastructure and high-quality borrower data; it is also sensitive to subjective judgements about recovery rates and strategic interactions between borrower and lender.

In the context of forestry carbon-sink pledge lending—where projects are long-lived, future carbon prices are highly uncertain and secondary market liquidity is low—the central research challenge is to integrate dynamic asset valuation (to capture price volatility and policy risk) with robust credit-risk measures (to reflect construction and operational uncertainties). A promising way forward lies in hybrid models that draw selectively on option-pricing to value volatility, on stress-testing to bound loss exposures and on default-probability analysis to anchor haircuts to borrower creditworthiness, thus balancing valuation precision, risk sensitivity and practical implementability.

### Value drivers for forest-based carbon assets

The valuation of forest-based carbon assets is driven chiefly by two interlinked factors: carbon price volatility, which determines the present value of future cash flows, and the volume of emissions reductions, which fixes the total tradable asset base and revenue potential. Secondary influences—from regional climate and growth cycles to project costs, regulatory frameworks, and market liquidity—shape the risk–return profile and must be incorporated into robust valuation.

To date, two broad methodologies have emerged. Expert judgement draws on practitioners’ qualitative assessments of local endowments (soil, topography, species), climatic trends, and forestry biology. Its strength lies in rapid, context-sensitive insights during project scoping, but it cannot assign precise weights to individual drivers or adapt easily as market conditions evolve. Data-driven analysis, by contrast, uses econometric tools—regression, structural equation models or numerical simulation—to estimate elasticities and probe causal pathways. This quantitative approach offers greater precision and scenario testing but hinges on high-quality, high-frequency data, stringent model assumptions, and intensive calibration.

In applied settings, expert judgement remains indispensable for early feasibility, while quantitative models underpin detailed risk management once projects mature. Yet both approaches struggle to capture the full dynamics of policy shifts, technological change, and market evolution over the multi-decadal life of forestry carbon assets. Nor do they readily accommodate the assets’ inherent characteristics of long duration, limited liquidity, and multiple revenue streams. Future research must therefore fuse qualitative expertise with quantitative rigor—leveraging scenario analysis, Monte Carlo simulation or real-options techniques—to dynamically model interactions among key value drivers across project lifecycles.

### Current landscape of pledge-based financing for forest-based carbon assets

Pledge-based lending linked to forest-based carbon assets represents a significant innovation in China’s green credit ecosystem. Under this model, enterprises pledge either verified or anticipated carbon reductions generated from forest-based carbon asset projects as collateral. The asset’s value is professionally appraised, and commercial banks extend credit commensurate with the assessed collateral, thereby supporting the operational and development needs of the underlying forestry projects.

Currently, the two principal forms of collateral accepted in pledge-based financing are: (1) verified forest-based carbon asset reductions, which refer to carbon units already generated, certified, and eligible for trading; and (2) anticipated carbon asset reductions, referring to future emissions reductions expected from registered but not yet credited forestry projects. In recent years, the volume and geographic reach of pledge-based lending in this area have grown steadily. According to publicly available data, such financing arrangements now span multiple provinces including Fujian, Zhejiang, Anhui, Yunnan, and Jiangsu (Table 1).

**Table 1:** Representative pledge-lending pilots by collateral type in China (verified and anticipated forest-based carbon reductions; pilot entities and launch years).

Collateral type	pilot entities (year)
Verified forest-based carbon reductions	Daxing’anling Tuqiang Forestry Bureau, Heilongjiang (2016); Fujian Jinshan Forest Resources Service Co., Ltd. (2021); Liancheng State-Owned Forest Farm, Longyan, Fujian (2021); Nanjing Tongzhou Gas Co., Ltd., Jiangsu (2023)
Anticipated forest-based carbon reductions	Jingxian State-Owned Forestry Development Co., Ltd., Xuancheng, Anhui (2021); Zhongnan Baicaoyuan Group Co., Ltd., Huzhou, Zhejiang (2021); Puer Kemaolinhua Forestry Chemical Co., Ltd., Yunnan (2022); Guangxi Jingui Pulp & Paper Co., Ltd. (2022); Zhangshan Forest Farm, Suqian Lakeside New Area, Jiangsu (2023)

From a policy perspective, the Chinese government has placed considerable emphasis on fostering the development of pledge-based financing for forest-based carbon assets. A series of policy measures and regulatory documents have been issued, aimed at enhancing the underlying legal framework, financial infrastructure, and operational capacity of the sector. These include explicit commitments to improve green finance standards and evaluation systems, and to develop diversified products such as green credit, green bonds, and green insurance—each of which plays a role in facilitating the monetisation and liquidity of carbon assets through pledging.

The implementation of the Administrative Measures for Carbon Emissions Trading (Trial) has established a regulatory foundation for treating forest-based carbon assets as tradeable property rights. This policy shift enables the market valuation of forestry-based emission reductions, which is a precondition for credible and scalable pledge-based financing mechanisms. These regulatory developments serve to: (1) clarify ownership structures and trading rules for carbon assets, thereby boosting investor and lender confidence; and (2) reduce project owners’ financing costs and institutional risk through targeted fiscal incentives.

Some local governments have introduced risk compensation schemes and interest subsidies for financial institutions engaging in forest-based carbon pledge lending^42^. These mechanisms help to mitigate exposure to default risk and encourage greater participation from commercial banks and regional financial platforms. The availability of such tools has been instrumental in lowering entry barriers for forestry operators and promoting broader adoption of carbon financing models.

From a growth perspective, the pledge-based financing market for forest-based carbon assets is expected to expand rapidly in line with national dual carbon targets and accelerating green finance policy rollouts. On the demand side, more forestry enterprises are recognising the economic value of their carbon assets and are increasingly willing to monetise them through structured finance. On the supply side, financial institutions are launching a variety of new instruments—such as lower interest rates, extended loan terms, and higher pledge ratios—thereby improving the scalability and accessibility of the market.

Geographically, however, development remains uneven. Coastal provinces with more advanced financial infrastructure—such as Fujian, Zhejiang, and Guangdong—are leading in terms of market size and product innovation. In these regions, pledge financing structures have evolved to include not only verified reductions and anticipated reduction rights, but also novel instruments such as carbon invoice pledging and forest carbon revenue pledging, integrated into broader local industrial ecosystems. The diversity of products and the maturity of the value chain reflect a well-established synergy between forestry sectors and carbon finance institutions.

By contrast, central and western regions, despite possessing rich forest resources, lag behind due to lower economic development levels, less developed financial services infrastructure, and weaker awareness among local stakeholders. Here, pledge-based financing models are relatively rudimentary—typically limited to conventional forestland mortgages or basic anticipated carbon revenue pledges. Innovation remains constrained, and stronger policy support and technical assistance are needed to unlock the region’s potential.

In sum, while China’s pledge-based financing ecosystem for forest-based carbon assets is gaining institutional traction and market credibility, regional disparities persist, and the sector’s future expansion will depend on continued regulatory innovation, cross-sectoral coordination, and the broader integration of carbon finance into the national climate governance framework.

## Results

### Model development: real-options and B-S valuation

This study develops a contract-consistent framework for pricing the financing value of forest-based carbon collateral under uncertainty. The framework distinguishes explicitly between present value and option value, but treats both within a lender-oriented collateral-pricing structure rather than as stand-alone asset-pricing objects. Present value is defined on the basis of the effectively deliverable collateral quantity, while option value captures the economically relevant timing flexibility embedded in delayed monetisation under uncertain market conditions.

The forecasting component is used to recover a disciplined forward-looking price environment from thin and irregular weekly CCER transactions, but it does not mechanically determine the strike. Instead, the strike is specified as a contract-grounded debt-coverage threshold derived from the observed loan structure. Once total economic value is obtained, the pledge-rate equation maps that value into a model-implied pledgeable amount under downside risk. Sensitivity analysis is then used to examine how financing capacity responds to changes in carbon price, effective deliverability, volatility identification and prudential threshold calibration. This framework therefore provides a contract-consistent and risk-adjusted method for quantifying both economic collateral value and the lower-bound lender-oriented pledgeable amount implied under stylised non-loss lending conditions.

Black–scholes option pricing model.

(1)(1)(1) Geometric Brownian motion assumption.

In financial theory, an option is a contract that grants the holder the right—but not the obligation—to buy or sell an underlying asset at a predetermined price within a specific time frame. This embedded optionality creates an asymmetry of obligations: the buyer holds a contingent claim, while the seller bears the associated exposure. Options may be classified as European (exercisable only at maturity) or American (exercisable at any time before expiry). These features render option pricing a core concern in both financial and real asset valuation contexts.

The Black–Scholes (B–S) model provides a foundational analytical framework for valuing European options, based on the assumption that asset prices follow a geometric Brownian motion (GBM). In this framework, market conditions are assumed to be frictionless and arbitrage-free, with continuous trading and perfect divisibility of assets. The GBM process assumes that the asset’s logarithmic returns are normally distributed, and price paths evolve continuously.

A standard Brownian motion, denoted $\left\{\mathrm{B}\left(\mathrm{t}\right),\mathrm{t}\ge 0\right\}$, is a continuous-time stochastic process defined on the non-negative real line. It is characterised by the following three properties:

1. $$\mathrm{B}\left(0\right)=0$$;
2. For any $0<\mathrm{n}<\mathrm{t},$ the increment $\mathrm{B}\left(\mathrm{t}\right)-\mathrm{B}\left(\mathrm{n}\right)$ follows a normal distribution with mean zero and variance $\mathrm{t}-\mathrm{n}$;
3. For any two non-overlapping intervals $\left\{{\left[n\right\}}_{i},{y}_{i}\right]$, the increments $\mathrm{B}\left({n}_{i}\right)$ and $\mathrm{B}\left({y}_{i}\right)$ are statistically independent.

When a linear drift term $\upmu t$ and a diffusion coefficient $\upsigma$ are added to the standard Brownian motion, the resulting process becomes a geometric Brownian motion (GBM). The infinitesimal form of a GBM is expressed as the following stochastic differential equation (SDE):

1 $$\mathrm{d}\mathrm{X}\left(\mathrm{t}\right)=\upmu \mathrm{d}\mathrm{t}+\upsigma \mathrm{d}\mathrm{B}\left(\mathrm{t}\right)$$

Here, $\mathrm{X}\left(t\right)$ denotes the return process of the underlying asset. If we let $\mathrm{S}\left(t\right)$ represent the market price of the forest-based carbon asset at time $\mathrm{t}$, then $\mathrm{d}\mathrm{S}\left(t\right)$ represents its infinitesimal change, and the instantaneous return is $\frac{dS\left(t\right)}{S\left(t\right)} .$ Substituting $\mathrm{X}\left(t\right)=\mathrm{ln}S\left(t\right)$ into formula (1) yields the familiar geometric Brownian motion for $\mathrm{S}\left(t\right),$ which characterises the stochastic evolution of the carbon price.

2 $$\mathrm{d}\mathrm{S}\left(\mathrm{t}\right)=\upmu \mathrm{S}\left(\mathrm{t}\right)\mathrm{d}\mathrm{t}+\upsigma \mathrm{S}\left(\mathrm{t}\right)\mathrm{d}\mathrm{B}\left(\mathrm{t}\right)$$

Thus, the price process $\mathrm{S}\left(t\right)$ of the carbon asset follows a GBM, where $\upmu$ is the expected annual return and $\upsigma$ is the annualised volatility. This stochastic process is formally classified as an Itô diffusion process, combining both deterministic drift and random fluctuation components.

To derive the closed-form solution for $\mathrm{S}\left(t\right)$, we apply Itô’s Lemma to the natural logarithm of the price process, letting $\mathrm{f}=\mathrm{ln}S\left(t\right)$. The differential form is:

3 $$d\left(\mathrm{ln}S\right)=df=\left(\mu -\frac{{\sigma }^{2}}{2}\right)dt+\sigma dB$$

Integrating both sides over the time horizon $\left[0, T\right]$, and then exponentiating the result, we obtain the explicit solution for the price at time $\mathrm{T}$:

4 $$S\left(T\right)=S\left(0\right)\mathrm{exp}\left[\left(\mu -\frac{{\sigma }^{2}}{2}\right)T+\sigma B\left(T\right)\right]$$

This formula expresses the evolution of the carbon asset price over time as a stochastic exponential process. It serves as the theoretical foundation for subsequent option pricing under the Black–Scholes framework.

(2)(2)(2) Valuation of forest-based carbon asset options using the black–scholes model.

To construct a risk-free portfolio, the value of the forestry carbon asset is combined with a corresponding option. In such a hedged position, both the price of the underlying asset and the value of the option are subject to fluctuations in market price, interest rate movements, and volatility. The core idea of this strategy is that, over a short time interval, the portfolio becomes effectively insulated from stochastic shocks. Under the assumption of no arbitrage, the return on this risk-free portfolio must be equal to the risk-free interest rate.

Given the standard assumptions of the Black–Scholes framework and based on the stochastic differential equation derived earlier, the value of a European call option on the forestry carbon asset is given by the classical Black–Scholes formula:

The value $\mathrm{C}$ of the option is defined as:

5 $$\mathrm{C}=\mathrm{S}\cdot \mathrm{N}\left({\mathrm{d}}_{1}\right)-\mathrm{X}\cdot {\mathrm{e}}^{-\mathrm{r}\mathrm{T}}\cdot \mathrm{N}\left({\mathrm{d}}_{2}\right)$$

where:

6 $${d}_{1}=\frac{\mathrm{ln}\left(S/X\right)+\left(r+\frac{1}{2}{\sigma }^{2}\right)T}{\sigma \sqrt{T}}$$

7 $${\mathrm{d}}_{2}={\mathrm{d}}_{1}-\upsigma \sqrt{\mathrm{T}}$$

And:

$\mathrm{C}$: value of the European call option.

$\mathrm{S}$: current market price of the forest-based carbon asset.

$\mathrm{X}$: exercise (strike) price of the option.

$\mathrm{r}$: risk-free interest rate.

$\upsigma$: annualised volatility of the carbon asset’s returns.

$\mathrm{T}$: time to maturity (in years).

$\mathrm{N}\left(\bullet \right)$: cumulative distribution function of the standard normal distribution.

To operationalise the option-pricing framework in this study, five parameters are required, but their interpretation follows the contract-consistent empirical design adopted in the manuscript.

First, ${S}_{0}$ is the observed spot price of the forest-based carbon asset at the valuation date.

Second, $X$ is not defined as a forecasted future carbon price, but as a contract-grounded monetisation threshold, specified as $X=\kappa {D}_{T}/{Q}_{eff}$, where ${D}_{T}={L}_{0}(1+R{)}^{T}$ denotes the repayment obligation at maturity and ${Q}_{eff}=\theta {Q}_{nom}$ denotes the effectively deliverable collateral quantity.

Third, $r$ is the risk-free interest rate corresponding to the contractual maturity term, proxied by the three-year Chinese government bond yield at the valuation date.

Fourth, $\sigma$ is the annualised volatility parameter, estimated from recent-window weekly gap-adjusted returns using a robust estimator, with a separate full pre-loan estimate retained as a high-volatility stress case.

Fifth, $T$ denotes the contractual maturity of the option-based valuation horizon and is aligned with the observed three-year loan term.

Within this framework, the CNN–LSTM forecasts are used to recover the forward-looking market environment relevant to monetisation and scenario design, but they do not mechanically determine the strike. This parameterisation aligns the Black–Scholes module with the observed financing structure and the thin-market characteristics of the CCER market.

### Forecasting forest-based carbon asset prices with CNN–LSTM

To improve the accuracy and robustness of price forecasting for forest-based carbon assets under market uncertainty, this study employs a hybrid deep learning architecture that integrates Convolutional Neural Networks (CNN) and Long Short-Term Memory (LSTM) networks. Compared with traditional econometric or time-series models, this architecture offers distinct advantages in capturing both localised volatility and long-range temporal dependencies that characterise carbon asset dynamics.

The CNN module is responsible for extracting salient features from the input time series by identifying short-term patterns using local connectivity and weight sharing. This allows the model to detect important fluctuations in asset price behaviour within narrow time windows. The LSTM component then models long-term dependencies and nonlinear interactions between time steps, which are critical for understanding the evolving behaviour of forest-based carbon asset prices. The combined structure enhances learning efficiency and supports generalisation even when working with relatively small datasets.

The model architecture consists of an input layer, a one-dimensional convolutional layer, an average pooling layer, an LSTM layer, and a fully connected output layer. The CNN component first applies one-dimensional convolution to extract local features from the univariate time series. This is followed by an average pooling operation that reduces dimensionality and mitigates overfitting, while preserving structural continuity. The LSTM layer then captures sequential dependencies in the feature representations, allowing for dynamic forecasting. Notably, the model also demonstrates strong error correction capabilities: if errors or distortions arise during the feed-forward process, the convolutional filters help reshape optimised feature vectors to minimise propagation of prediction errors downstream.

The CNN–LSTM framework is particularly well-suited for capturing the nonlinear, partially structured nature of carbon asset price data. Unlike traditional forecasting methods that often depend on extensive historical inputs, this hybrid model delivers high predictive accuracy with a relatively limited data footprint. Additionally, parallel computation in the CNN layer and efficient sequential modelling in the LSTM layer reduce computational complexity while improving scalability.

The mathematical operation of the convolutional transformation is expressed as:

8 $$\mathrm{C}=\upsigma \left(\mathrm{W}\odot \mathrm{X}+\mathrm{b}\right)$$

where:

$\mathrm{C}$ is the output feature map,

$\upsigma$ is the activation function,

$\mathrm{W}$ is the convolutional kernel (weight matrix),

$\mathrm{X}$ is the input time series of forest-based carbon asset prices,

$\mathrm{b}$ is the bias term,

$\odot$ denotes the convolution operation.

To evaluate model performance, two standard metrics are adopted: the Root Mean Square Error (RMSE) and the Mean Absolute Percentage Error (MAPE). These indicators allow for a robust assessment of both the magnitude and proportion of forecast error.

The RMSE is defined as:

9 $$RMSE=\sqrt{\frac{1}{N}\sum_{c=1}^{N}{\left({y}_{c}-\widehat{{y}_{c}}\right)}^{2}}$$

where:

${y}_{c}$ is the observed value of the ${c}^{th}$ sample,

$\widehat{{y}_{c}}$ is the predicted value of the ${c}^{th}$ sample,

$\mathrm{N}$ is the total number of observations.

RMSE values lie in the interval $\left[0, +\infty \right]$; smaller values indicate greater prediction accuracy and less error variance.

The MAPE quantifies average proportional error across the prediction horizon:

10 $$\mathrm{M}\mathrm{A}\mathrm{P}\mathrm{E}=\frac{100\mathrm{\%}}{\mathrm{N}}{\sum}_{\mathrm{c}=1}^{\mathrm{N}}\left|\frac{{\mathrm{y}}_{\mathrm{c}}-\widehat{{\mathrm{y}}_{\mathrm{c}}}}{{\mathrm{y}}_{\mathrm{c}}}\right|$$

where all terms are as defined above. MAPE values also lie within $\left[0, +\infty \right]$, and interpretability is intuitive: a MAPE below 10% typically indicates high accuracy, 10–20% suggests reasonable but improvable performance, while values above 20% imply that model recalibration or architectural refinement may be necessary.

By combining a dual-path feature learning strategy with rigorous evaluation metrics, this CNN–LSTM framework provides a flexible, accurate, and computationally efficient tool for forecasting the price of forest-based carbon assets. It serves as a key methodological component in the broader valuation of carbon-linked financial instruments such as real-options-based pledge financing.

### Valuation of forest-based carbon asset projects

Within the contract-consistent framework adopted in this study, the total economic value of a forest-based carbon asset project is defined as the sum of a present-value component and an option-value component. The present-value component reflects the immediately recognisable collateral value at the valuation date, while the option-value component captures the timing flexibility associated with deferring monetisation under uncertain future market conditions.

A key feature of this framework is that valuation is based not on the full nominal project volume, but on the effectively deliverable collateral quantity. Let ${Q}_{nom}$ denote the nominal carbon quantity and let $\theta$ denote the deliverable credit fraction. The lender-relevant collateral quantity is then defined as:

11 $${\mathrm{Q}}_{\mathrm{e}\mathrm{f}\mathrm{f}}=\uptheta {\mathrm{Q}}_{\mathrm{n}\mathrm{o}\mathrm{m}}$$

where $\theta$ captures prudential reductions associated with MRV uncertainty, verification delay, permanence risk, reversal risk, and related implementation frictions. This definition ensures that the valuation framework is aligned with the amount of carbon-related value that can credibly be recognised for financing purposes, rather than with the full nominal project volume alone.

The present value of the project is therefore given by

12 $$PV={S}_{0}{Q}_{eff}$$

where ${S}_{0}$ is the observed current market price of the forest-based carbon asset at the valuation date.

The option component is valued using a Black–Scholes framework in which the strike is specified as a contract-grounded monetisation threshold rather than as a forecasted future carbon price. Let ${L}_{0}$ denote the observed loan principal, $R$ the contractual borrowing rate, and $T$ the contractual maturity. Under an annual compounding convention, the repayment obligation at maturity is defined as ${D}_{T}={L}_{0}(1+R{)}^{T}$. The strike is then specified as a unit debt-coverage threshold:

13 $$\begin{array}{cccc}& X=\kappa \frac{{D}_{T}}{{Q}_{eff}}& & \end{array}$$

where $\kappa \ge 1$ is a prudential coverage factor. This formulation ties the strike directly to the observed financing structure and reflects the price level at which monetisation becomes rational from the perspective of debt coverage.

Given ${S}_{0}$, $X$, $r$, $\sigma$, and $T$, the per-unit option value $c$ is obtained from the Black–Scholes call formula, and the total option value of the pledged carbon-related asset is given by

14 $$\begin{array}{cccc}& CV=c{Q}_{eff}& & \end{array}$$

The total economic value of the pledged carbon-related asset is then defined as

15 $$\begin{array}{cccc}& TV=PV+CV& & \end{array}$$

This decomposition distinguishes two analytically different objects within the collateral-pricing problem. The first is the immediately recognisable value of the effectively deliverable carbon-related asset at the valuation date. The second is the additional value associated with waiting under uncertain future market conditions, conditional on the contract-grounded monetisation threshold.

This formulation is deliberately different from a simple forecast-discount approach. In principle, one could discount a forecasted future carbon price back to the valuation date to obtain an expected-value benchmark. However, such an approach would remain a linear expected-value proxy and would not capture the asymmetric exposure that defines pledge-based financing. In the lending context, downside risk arises when collateral value falls below the repayment obligation, so valuation must reflect not only the expected price level but also the distribution of uncertainty and the resulting convex payoff structure. Accordingly, the CNN–LSTM forecasting module is used to recover a forward-looking market environment relevant to valuation, whereas the option-pricing structure captures the economically relevant non-linearity under uncertainty. The total economic value $TV$ therefore represents a contract-consistent collateral valuation benchmark, which is translated into lender-relevant financing capacity only in the subsequent pledge-rate stage.

### Construction of a pledge rate model based on the black–scholes framework

Drawing on current pledge-financing practices for forest-based carbon assets in China, this study develops an analytical pledge-rate model calibrated to the core observable terms of the Ning’er case. Publicly available disclosures identify a three-year loan contract with a fixed annual interest rate of 3.65%, backed by the expected revenue rights associated with the underlying forest-based carbon asset project. As the full contract text is not publicly available, the model does not attempt to reproduce every legal and operational clause of the transaction. Instead, it adopts transparent simplifying assumptions for non-public elements of the cash-flow structure. Specifically, the loan is modelled as a fixed-rate bullet structure in which principal and accrued interest are repaid at maturity, and no prepayment is assumed. These assumptions are intended to support a contract-consistent empirical valuation framework rather than a contract-exhaustive reconstruction of the underlying transaction, and are reported explicitly in the table summarising the observed contract terms and modelling assumptions.

The pledge-rate model is therefore anchored to the principal observable features of the actual loan, namely the loan amount, contractual maturity, interest rate, and collateral basis, while treating unobserved contractual details, such as repayment timing conventions and prepayment provisions, through explicit and transparent modelling assumptions. This treatment is intended to preserve comparability between the model-implied pledge value and the observed loan, while avoiding stronger claims than the available contractual information can support.

Let $r$ denote the continuously compounded risk-free rate, $T$ the loan maturity, $R$ the borrowing rate, $w$ the pledge rate, ${P}_{0}$ the current unit price of the pledged carbon-related asset, ${P}_{T}$ its unit price at maturity, and $Q$ the pledged quantity. The total collateral value at origination is defined as

16 $$\begin{array}{cccc}& {C}_{0}={P}_{0}Q& & \end{array}$$

The loan amount received by the borrower at origination is

17 $${L}_{0}=w{C}_{0}$$

At maturity, the market value of the pledged carbon-related asset is

18 $${C}_{T}={P}_{T}Q$$

For analytical tractability in the pledge-rate derivation, the lender-side repayment obligation is expressed in continuous-compounding form as

19 $${K}_{p}={L}_{0}{e}^{RT}=w{C}_{0}{e}^{RT}$$

where ${K}_{p}$ denotes the lender-side strike. This notation is used here to distinguish the repayment-related strike in the pledge-rate equation from the monetisation threshold $X$ defined in the collateral valuation module.

Two possible cases arise at maturity. If the terminal collateral value is at least as large as the repayment obligation, the borrower has an incentive to repay in full and the lender incurs no loss:

20 $$\mathrm{L}\mathrm{o}\mathrm{s}\mathrm{s}(T)=0, \text{if }{C}_{T}\ge {K}_{p}$$

If the terminal collateral value falls below the repayment obligation, the lender bears a default loss equal to the shortfall:

21 $$\mathrm{L}\mathrm{o}\mathrm{s}\mathrm{s}(T)={K}_{p}-{C}_{T}, \text{if }{C}_{T}<{K}_{p}$$

From the lender’s perspective, this arrangement is economically equivalent to a short European put written on the pledged asset, with strike ${K}_{p}$. The value of the implicit put option is therefore given by

22 $$\Pi ={K}_{p}{e}^{-rT}N(-{d}_{2})-{C}_{0}N(-{d}_{1})$$

where

23 $${d}_{1}=\frac{\mathrm{ln}\left(\frac{{C}_{0}}{{K}_{p}}\right)+\left(r+\frac{{\sigma }^{2}}{2}\right)T}{\sigma \sqrt{T}}=\frac{-\mathrm{l}\mathrm{n}w+\left(\frac{{\sigma }^{2}}{2}-R+r\right)T}{\sigma \sqrt{T}}$$

and

24 $${d}_{2}={d}_{1}-\sigma \sqrt{T}$$

Referring to the general pledge-rate model, the pledge rate must satisfy the lender’s break-even condition:

25 $$w{C}_{0}{e}^{(R-r)T}-\Pi =w{C}_{0}$$

Substituting Eq. (22) into Eq. (25), and applying the identity $N(x)+N(-x)=1$, yields

26 $$w{e}^{(R-r)T}N({d}_{2})+N(-{d}_{1})=w$$

which can be rearranged as the pledge-rate decision equation

27 $$\begin{array}{cccc}& {e}^{(R-r)T}N({d}_{2})=1-\frac{1}{w}N(-{d}_{1})& & \end{array}$$

This is a nonlinear algebraic equation in $w$, solvable via numerical methods. It shows that the pledge rate is determined jointly by the interest-rate spread $\left(R-r\right)$, the loan term $T$, and the volatility $\sigma$ of the carbon asset price.

To examine the empirical implications of the framework, the subsequent sensitivity analysis varies four dimensions: carbon price, effective deliverability, recent-window volatility identification, and prudential threshold calibration. This design is intended to evaluate not only how collateral value responds to market conditions, but also how much of that value remains translatable into lender-recognised pledgeable capacity. The resulting evidence provides theoretical guidance for policymakers and financial institutions seeking to improve the capitalisation of forest-based carbon assets and support climate-aligned lending strategies.

## Empirical test of the pledge valuation model: a case study of forestry carbon asset pledge financing in Yunnan, China

This case study focuses on the first pledge-based loan in Yunnan Province backed by the expected revenue rights of a forestry carbon asset project. The aim is to assess the valuation logic and practical implementation of the pledge financing model applied. The selected project, hereafter referred to as the Ning’er Reforestation Carbon Sink Project, is operated by Pu’er Kemaolinhua Forestry and Chemical Company, a regional forestry enterprise engaged in reforestation for carbon credit generation.

The project is located in three administrative areas—De’an Township, Meizi Township, and Dehua Township—within the Ning’er Hani and Yi Autonomous County, Yunnan Province. It was implemented under the direct operational responsibility of Pu’er Kemaolinhua. The project holds ecological and socio-economic importance in several respects. First, by establishing scientifically planned reforested plots, it facilitates measurable carbon sequestration from the atmosphere and contributes to scaling up forestry-based emission reductions. Second, the project enhances the integrity of local ecosystems through forest rehabilitation, soil and water conservation, and biodiversity preservation. Finally, it provides income-generating opportunities for local farmers and contributes to the sustainable development of the surrounding rural economy.

In September 2022, a joint evaluation was carried out by the Agricultural Bank of China (ABC), Ning’er Branch, in collaboration with the Ning’er County Government and related stakeholders. A hybrid assessment method combining on-site inspections and document-based technical analysis confirmed that the project covered a total of 51,365 mu (approximately 3,424 hectares) and was projected to generate 719,680 tonnes of forest-based carbon asset stock over its crediting period.

Using a benchmark CCER (China Certified Emission Reduction) spot price of CNY 60 per tonne on the pledge date, the total estimated value of the pledged carbon asset was CNY 43.18 million. Based on this valuation, the Agricultural Bank of China issued a pledge-backed loan of CNY 12 million, yielding a pledge ratio of 27.79%. The credit agreement stipulated a term of three years at an annual interest rate of 3.65%.

For the empirical application, valuation is aligned with the core observable terms of the actual loan contract rather than with a simplified one-year modelling horizon. The loan is treated as originating in September 2022, with a contractual maturity of three years and an annual interest rate of 3.65%, consistent with publicly available information on the observed transaction. The forecasting module is trained on pre-loan weekly transaction data and targets the future 4-week average CCER price, which is used to characterise the forward-looking market environment relevant to valuation. By contrast, the option maturity and pledge-valuation horizon remain aligned with the observed three-year contract term, so that the model-implied pledge value is comparable to the observed loan on a like-for-like basis. Because the full loan contract is not publicly available, the empirical application does not attempt to reproduce every contractual clause in detail. Instead, it is anchored to the core observable loan terms, including the loan amount, maturity, interest rate, collateral basis and loan-side appraisal basis, while non-public contractual details are handled through transparent modelling assumptions reported explicitly in the manuscript.

To improve transparency, Table 2 distinguishes observed contract terms from modelling assumptions. Observable terms include the loan amount, contractual maturity, stated interest rate, project scale, and the pledge of expected revenue rights associated with the forestry carbon asset project. By contrast, non-public details—such as the exact repayment schedule, prepayment provisions, and any dynamic revaluation arrangements—are not directly observable from public sources and are therefore treated through clearly stated simplifying assumptions. This distinction allows the empirical application to remain institutionally grounded without overstating the degree of contractual precision available in the public domain.

**Table 2:** Observed contract terms and modelling assumptions.

Item	Observed contract term	Modelling assumption in this study
loan origination date	September 2022	Valuation is anchored to 30 September 2022
loan maturity	3 years	3 years
interest rate	3.65%	3.65%
loan amount	RMB 12 million	RMB 12 million
collateral basis	Future forestry carbon-sink income pledge + forest-tenure mortgage	Financing valuation is anchored to the loan-side appraisal basis rather than the full PDD project volume
valuation date	—	30 September 2022
forecasting target	—	Future 4-week average price constructed from weekly transaction data and used to characterise the forward-looking market environment relevant to valuation
option maturity	—	3 years
deliverability treatment	—	${Q}_{eff}=\theta {Q}_{nom}$, with $\theta =0.90$ in the baseline specification
strike definition		$X=\kappa {D}_{T}/{Q}_{eff}$, where ${D}_{T}={L}_{0}{\left(1+R\right)}^{T}$ and $\kappa =3.6$ in the baseline specification
cash-flow assumption	—	Bullet repayment: principal and interest are paid in full at maturity

Note: Observed loan amount, tenor, interest rate and collateral structure are taken from public financing disclosures for the Ning’er case. Those disclosures also report a loan-side appraisal basis of 51,365 mu, 719,680 tCO_2_e and an indicative value of RMB 43.18 million at RMB 60/t. The PDD, by contrast, describes the underlying afforestation project as 47,848 mu, with a 30-year crediting period and 1,100,038.4 tCO_2_e of expected reductions. The baseline framework therefore uses the loan-side appraisal basis for financing valuation, while the PDD is used only to characterise the broader asset background, including ownership, crediting duration and permanence treatment. Forecasting is implemented through a weekly future 4-week average CCER price target rather than through a three-year maturity price forecast. Option maturity and collateral valuation remain aligned with the observed three-year loan structure.

Table 2 separates observed contract terms from modelling choices introduced to align valuation with the actual financing structure. The public case record identifies a RMB 12 million loan with a three-year tenor, an interest rate of 3.65%, and a collateral structure combining future forestry carbon-sink income pledge with forest-tenure mortgage. Because the public disclosures do not report a contractual valuation date or repayment schedule, the baseline model fixes the valuation date at 30 September 2022, aligns option maturity with the observed three-year loan term, adopts a bullet repayment structure under which principal and interest are settled at maturity, and uses a weekly future 4-week average CCER price target to characterise the forward-looking market environment relevant to valuation. To maintain consistency between the financing case and the underlying carbon asset, valuation is based on the loan-side appraisal basis reported in the financing disclosures, whereas the PDD is used only to define asset ownership, crediting duration and permanence treatment.

### Forecasting future CCER prices using the CNN–LSTM model

For the purposes of this study, MATLAB 2024a was selected as the modelling platform for data processing and pledge-based valuation simulations. Its integrated Deep Learning Toolbox provides a streamlined environment for developing hybrid CNN–LSTM networks. In addition, MATLAB’s high-performance numerical computation capabilities enable efficient training of deep learning models and rapid processing of time-series data, thereby improving both modelling accuracy and execution speed.

The China Certified Emission Reduction (CCER) transaction price is adopted in this study as a proxy for the market value of forest-based carbon assets. Among available trading platforms, the Sichuan United Environment Exchange (SUEE) was selected as the primary data source because it provides relatively transparent and comparatively continuous public transaction disclosures over the period relevant to the Ning’er financing case. However, the forecasting module is not designed as a stand-alone high-frequency prediction exercise. Rather, it is constructed to generate a forward-looking price input for the broader pledge-valuation framework under thin-market conditions.

The institutional evolution of the CCER market is central to interpreting the underlying price series. After the suspension of new CCER project registration in March 2017, the market remained closed to new project approvals for nearly seven years, with only previously registered legacy projects continuing to circulate. During 2023, the regulatory framework for the national voluntary carbon market was gradually rebuilt, and the national CCER market was formally relaunched on 22 January 2024. Because the observed loan contract spans both the prolonged suspension phase and the initial stage of market restart, transaction activity over the relevant period is characterised by intermittent trading, uneven liquidity and segmented price formation, rather than by a continuous and homogeneous market process. These features imply that the forecasting problem is not simply one of extrapolating a dense time series, but one of extracting a valuation-relevant forward signal from an irregular and structurally discontinuous market record.

Against this background, the final empirical specification uses weekly transaction data rather than monthly observations. This choice is methodologically preferable because weekly data retain substantially more of the market information available around the loan origination date and are better able to capture short-term price movements that monthly aggregation would smooth away. Such temporal granularity is particularly important in this study, where the pricing inputs need to reflect market conditions as closely as possible at the time the pledge contract was formed. At the same time, the weekly series is handled conservatively: missing observations are not mechanically interpolated into an artificial continuous price path, and model-ready samples are constructed only when both the target value and the required lagged inputs are adequately observed. This approach helps preserve market realism and avoids overstating the informational content of a thinly traded market series.

To preserve consistency with the information that would have been available at the time the financing decision was made, the CNN–LSTM forecasting model is trained and calibrated exclusively on the pre-loan weekly sample spanning 31 August 2018 to 30 September 2022. The subsequent period, from 14 October 2022 to 3 January 2025, is reserved strictly for out-of-sample evaluation. This sample split mirrors the actual timing of the three-year loan contract and ensures that the valuation framework relies only on information observable at loan origination.

The forecasting target is defined as the future 4-week average CCER price, measured as the average of observed transaction prices over the subsequent four weeks, rather than as a single weekly point price. This choice is justified on both empirical and economic grounds. Empirically, single-week prices in the CCER market are highly sensitive to sparse trading, localised transaction concentration and short-lived spikes, which makes them an unstable basis for collateral valuation. Economically, pledge-based financing depends less on the exact level of an individual transaction than on the forward price environment relevant to the collateral’s expected liquidation value. The future 4-week average therefore provides a more stable and valuation-relevant forecasting target than raw weekly point prices.

The model is constructed using a compact weekly feature set derived entirely from information available up to the forecast origin. These inputs include the log-transformed current price, recent return dynamics, short rolling means, transaction volume, transaction value, low-volume indicators, imputation flags, and the elapsed time since the previous observed transaction price. Their function is not to create an excessively flexible predictive structure, but to provide the CNN–LSTM with sufficient contextual information to distinguish routine price-platform behaviour from episodes of repricing stress. In this sense, the forecasting module remains explicitly lender-oriented: its purpose is to recover a disciplined forward price input under uncertainty rather than to fit every short-lived fluctuation in the historical series.

The weekly CNN–LSTM is therefore treated as a conservative, valuation-oriented forecasting tool. Its performance is assessed in two complementary ways. First, rolling-origin validation is applied within the pre-loan sample to examine stability across alternative historical windows. Second, the model is evaluated against a naive 4-week carry-forward benchmark in the genuine post-loan holdout period. This benchmark comparison is essential because it shows whether the CNN–LSTM provides information beyond simple price persistence. As shown below, this forecasting design yields stronger robustness when the prediction task is aligned with the future 4-week average price, which is more directly relevant for option-based collateral valuation.

**Fig. 1:**
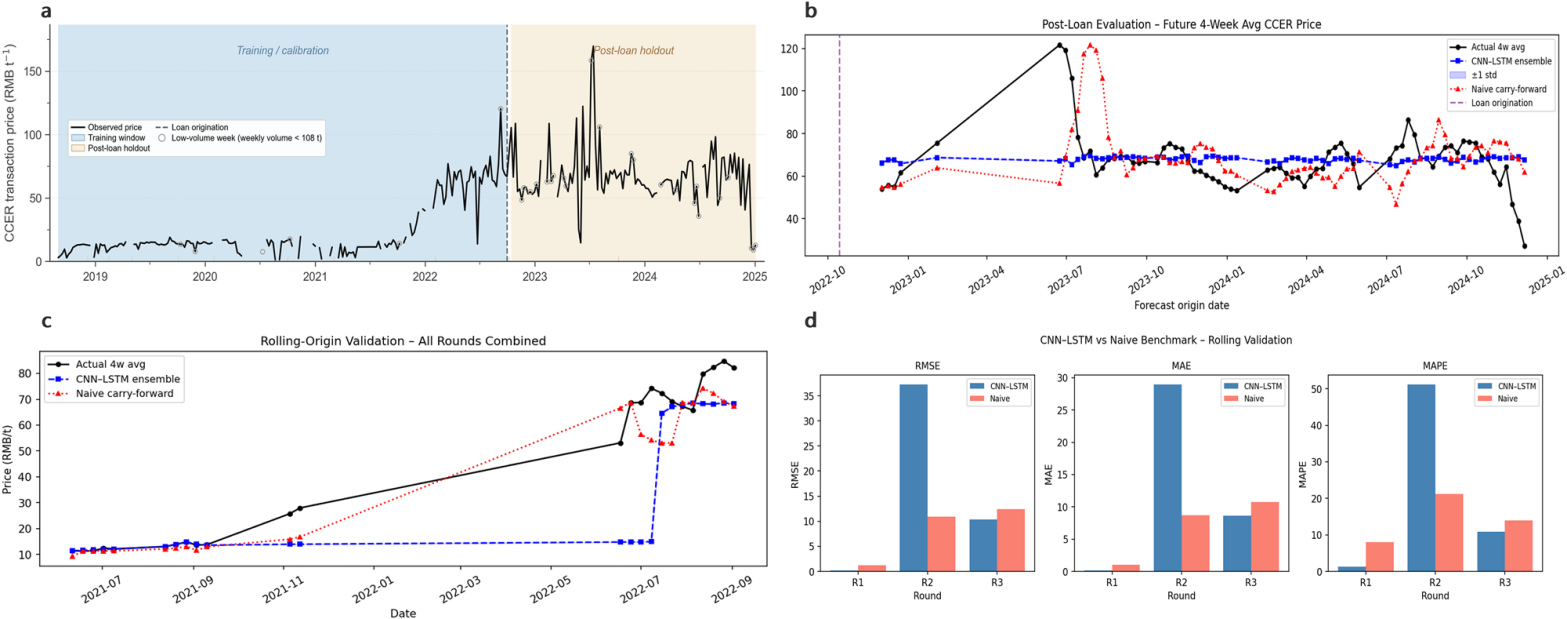
Weekly CNN-LSTM forecasting design and main results for the future 4-week average CCER price. (**a**) Weekly CCER transaction prices over the study period, with the training/calibration window and post-loan holdout window indicated. (**b**) Actual and predicted future 4-week average prices in the post-loan holdout, comparing the CNN-LSTM ensemble with the naive benchmark. (**c**) Rolling-origin validation results for the future 4-week average target. (**d**) Summary comparison of CNN-LSTM and naive benchmark performance across rolling validation and post-loan holdout.

Figure 1 summarises the forecasting design and headline results. Panel a shows the weekly CCER transaction-price series over the full sample, marking the training/calibration window, the loan-origination cutoff and the post-loan holdout used for strict out-of-sample evaluation. Panel b compares realised and forecasted future 4-week average prices in the holdout period, providing a direct test of model performance against the naive benchmark. Panel c reports rolling-origin validation across repeated forecasting rounds, and panel d condenses the comparison using RMSE, MAE and MAPE. Together, these panels situate the forecasting exercise in its contractual time structure and show that model performance should be evaluated not only by pointwise fit, but also by its stability across forecasting rounds and its accuracy relative to a simple alternative.

### Configuration of the CNN–LSTM forecasting model

The CNN–LSTM forecasting model adopts a lightweight weekly architecture tailored to the future 4-week average price target defined above. Its purpose is not to maximise architectural complexity, but to generate a stable and valuation-relevant forward price input under thin-market conditions. The model is therefore intentionally parsimonious, comprising a one-dimensional convolutional layer, a single LSTM layer, and a fully connected output layer. This structure is sufficient to capture local temporal patterns and sequential dependencies in the weekly price series, while limiting the parameter burden associated with a relatively small and irregularly observed training sample.

The CNN component functions as a local feature extractor. It operates on rolling weekly input windows and identifies short-horizon patterns in recent price and market-state variables, including transaction-price movements, return dynamics, and liquidity-related indicators. The convolutional layer is followed by a nonlinear activation function, after which the extracted features are passed to the recurrent module. The modelling strategy is deliberately conservative: it preserves the core CNN–LSTM logic while avoiding additional components that would increase fitting capacity without clear justification under the available sample conditions.

The LSTM layer captures the temporal dependence embedded in the weekly feature sequence. Its role is to summarise how recent price levels, short-run momentum, and market-state conditions jointly shape the expected future 4-week average price. The output from the final time step is then mapped by a fully connected layer into a one-dimensional forecast. Because the target is defined as a smoothed forward average rather than a single point price, the network is not required to reproduce every short-lived spike in the transaction record. Instead, it is trained to recover the medium-term price level that is more relevant for valuation. This task definition strengthens the alignment between the network architecture and the economic objective of the paper.

Three additional design features are central to this modelling framework. First, the model is trained on the log-transformed target so that scale differences at higher price levels do not dominate the optimisation process. Second, Huber loss is used in place of a purely quadratic loss function in order to reduce sensitivity to isolated spikes and outliers. Third, the training loss is recency-weighted, such that observations closer to the loan origination date receive greater weight than older observations drawn from a more weakly connected market regime. To further reduce small-sample instability, the model is re-estimated under five different random seeds, and the ensemble mean forecast is used as the main output. This ensemble procedure is best understood as a stabilisation device rather than a source of additional structural complexity.

Taken together, this CNN–LSTM configuration is best understood as a lightweight, lender-oriented forecasting tool. Its role is to transform an irregular weekly transaction record into a disciplined forward-looking price input for the broader option-based pledge valuation framework. The model is therefore assessed primarily by its ability to generate robust future price levels under realistic out-of-sample conditions, rather than by its ability to replicate every feature of the historical weekly price path.

### Model parameter configuration and forecast evaluation

## Main results

To generate forward-looking price inputs under thin-market weekly CCER conditions, the forecasting task is defined as the prediction of the future 4-week average CCER price, rather than a single weekly transaction price. The model combines a lightweight CNN–LSTM architecture with Huber loss, recency weighting and five-seed ensemble averaging. This setup is intended to recover a valuation-relevant forward price level while limiting sensitivity to sparse trading, local transaction concentration and short-lived spikes.

In the true post-loan holdout period, the CNN–LSTM outperforms a naive 4-week carry-forward benchmark across all four evaluation metrics. The CNN–LSTM achieves an RMSE of 13.87, an MAE of 8.91, a MAPE of 14.29% and an R^2^ of − 0.04, compared with 19.03, 12.88, 19.77% and − 0.95 for the naive benchmark, respectively. As shown in panel b-d in Fig. 1 above, the model captures the medium-term price level of the post-loan period more effectively than the benchmark, even though it does not reproduce every short-lived market fluctuation. The ratio of predicted to observed standard deviation reaches 0.87, indicating that the model preserves substantial variation in the target series rather than collapsing into a near-flat prediction path.

These results suggest that forecasting the future 4-week average price provides a more useful and more defensible forward input for valuation than forecasting raw weekly point prices. The resulting price target is better aligned with the collateral-pricing problem addressed in this study, because it reflects the short-horizon forward price environment relevant to asset valuation while filtering out part of the idiosyncratic noise generated by thin and discontinuous trading.

Rolling-origin validation nevertheless reveals one clear area of fragility. Average validation performance is strongly influenced by a single rally-driven regime-break window in which the market undergoes rapid upward repricing and the CNN–LSTM remains more conservative than the realised path. As shown in panel c-d in Fig. 1, this weakness is not distributed uniformly across all windows; rather, it is concentrated in episodes where momentum continuation is unusually strong. The forecasting problem is therefore best characterised not as one of general model breakdown, but as one of regime dependence.

## Robustness checks

To examine whether the forecasting module could be strengthened further, the CNN–LSTM forecasts were combined with the naive benchmark through a series of blending exercises. A first robustness test imposed a fixed blending weight of the form

28 $${\widehat{y}}_{t}=\upalpha {\widehat{y}}_{t}^{\mathrm{C}\mathrm{N}\mathrm{N}}+\left(1-\upalpha \right){\widehat{y}}_{t}^{\mathrm{n}\mathrm{a}\mathrm{i}\mathrm{v}\mathrm{e}}$$

with $\upalpha$ selected by rolling-validation RMSE. The resulting alpha grid search showed an almost monotonic shift in favour of the naive benchmark, and the selected solution converged to $\upalpha =0$, that is, the pure naive forecast. This outcome was driven by the rally-driven validation window, where momentum continuation dominated and heavily penalised the more conservative CNN–LSTM. However, the same fixed-weight solution performed poorly in the post-loan holdout, where the pure CNN–LSTM remained clearly superior. This result indicates that a single global blending weight cannot reconcile the heterogeneous forecasting regimes present in the sample.

**Fig. 2:**
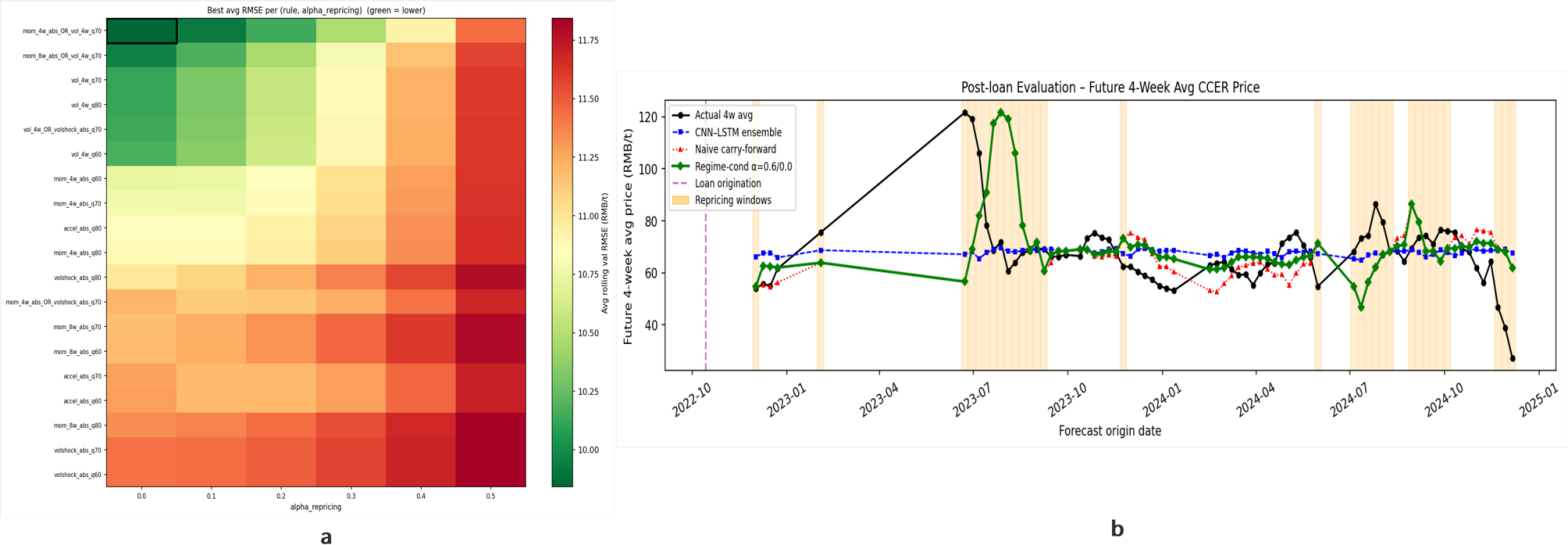
Robustness analysis of regime-dependent forecast combination for the future 4-week average CCER price. (**a**) Heatmap of average rolling-validation RMSE across candidate regime-classification rules and repricing-state blending weights. Lower RMSE indicates better validation performance. Candidate rules are based on threshold exceedances in recent momentum and short-run volatility, as defined from the training sample. The highlighted cell denotes the selected rule, which classifies a forecast origin as a repricing regime when either the absolute 4-week log-price change or the 4-week return volatility exceeds its 70th-percentile training threshold, and assigns ${\alpha}_{\mathrm{s}\mathrm{t}\mathrm{a}\mathrm{b}\mathrm{l}\mathrm{e}}=0.6$ and ${\alpha}_{\mathrm{r}\mathrm{e}\mathrm{p}\mathrm{r}\mathrm{i}\mathrm{c}\mathrm{i}\mathrm{n}\mathrm{g}}=0.0.$(**b**) Post-loan holdout evaluation of the regime-conditional blending strategy. The black line denotes the observed future 4-week average CCER price; the blue dashed line denotes the CNN-LSTM ensemble forecast; the red dotted line denotes the naive 4-week carry-forward benchmark; and the green line denotes the regime-conditional blended forecast. Amber shading marks forecast origins classified as repricing regimes under the selected rule. Although regime-conditional blending reduces forecast error in the rally-driven validation window, it does not outperform the baseline CNN-LSTM in the valuation-relevant post-loan holdout.

A second robustness test introduced regime-conditional blending, allowing the CNN–LSTM and naive forecasts to receive different weights depending on market state. The best-performing rule classified a forecast origin as repricing when either the absolute 4-week momentum or short-run volatility exceeded its 70th-percentile training threshold, and applied ${\upalpha}_{\mathrm{s}\mathrm{t}\mathrm{a}\mathrm{b}\mathrm{l}\mathrm{e}}=0.6$ and ${\upalpha}_{\mathrm{r}\mathrm{e}\mathrm{p}\mathrm{r}\mathrm{i}\mathrm{c}\mathrm{i}\mathrm{n}\mathrm{g}}=0.0$. This specification substantially reduced the error in the most difficult validation window, lowering the round-2 RMSE from 37.28 under the pure CNN–LSTM to 15.96, and improved average validation performance relative to the pure CNN–LSTM. However, it did not outperform the naive benchmark in validation overall, nor did it surpass the CNN–LSTM in the valuation-relevant post-loan holdout. In the post-loan sample, the regime-conditional specification achieved an RMSE of 18.67, which was slightly better than the fixed-$\upalpha$ naive solution but still materially worse than the CNN–LSTM. These results are reported in Fig. 2.

Taken together, the robustness checks clarify the structure of the forecasting problem rather than overturning the main result. They show that CCER price dynamics are strongly regime-dependent: rally windows reward a momentum-following rule, whereas the post-loan period rewards the more conservative, level-oriented behaviour learned by the CNN–LSTM. The evidence therefore supports the use of the CNN–LSTM as the main forecasting model for valuation, while the blending exercises help define the market conditions under which its performance is most reliable.

The weekly CNN–LSTM forecasts are used to characterise the forward-looking price environment relevant to collateral valuation under thin-market conditions. They do not mechanically determine the strike price in the Black–Scholes module. Instead, the option is defined as the project owner’s right to defer monetisation of the pledged carbon-related revenue right until market conditions justify sale at the contract-relevant monetisation window.

### Parameter determination and pledge financing valuation results

The valuation exercise is anchored to the observed loan structure. The Ning’er transaction is treated as originating on 30 September 2022, with a contractual maturity of three years and a fixed annual borrowing rate of 3.65%. Consistent with the loan-side appraisal basis disclosed publicly, the baseline nominal carbon quantity is 719,680 tCO₂e and the baseline market price at the pledge date is CNY 60 per tCO₂e. Because the collateral consists of expected carbon-related revenue rights rather than already deliverable spot units, the quantity entering the valuation is defined as the effectively deliverable collateral quantity rather than the nominal project volume alone:

29 $${Q}_{\mathrm{e}\mathrm{f}\mathrm{f}} =\theta {Q}_{\mathrm{n}\mathrm{o}\mathrm{m}}$$

where $\vdash \uptheta \in \left(\mathrm{0,1}\right]$ denotes the deliverable credit fraction. This term captures the prudential effects of MRV uncertainty, verification delay, permanence risk, reversal risk, and other implementation frictions that may reduce the volume effectively realisable for lending purposes. In the baseline specification, $\uptheta$ is set at 0.90 as a conservative modelling assumption, so that delivery-related risk enters the valuation framework directly rather than remaining only a qualitative consideration.

Within this framework, the Black–Scholes module values the owner’s flexibility to defer monetisation until the contract-relevant monetisation window. The strike is therefore specified as a unit monetisation threshold rather than as a network forecast. Let ${L}_{0}$ denote the observed loan principal, $\mathrm{R}$ the contractual borrowing rate, and $\mathrm{T}$ the contractual maturity. Under an annual compounding convention, the repayment obligation at maturity is given by

30 $${D}_{T}={L}_{0}{\left(1+\mathrm{R}\right)}^{T}$$

The unit monetisation threshold is then defined as

31 $$\mathrm{X}=\upkappa \frac{{D}_{T}}{{Q}_{\mathrm{e}\mathrm{f}\mathrm{f}}}$$

where $\upkappa \ge 1$ is a prudential coverage factor. When $\upkappa =1$, the threshold corresponds exactly to the per-unit price required to cover the contractual repayment obligation at maturity. When $\upkappa>1$, it incorporates an additional conservative buffer for monetisation costs, liquidation discounts, and covenant protection. In the baseline specification, $\upkappa$ is calibrated at 3.6. This value is not imposed mechanically, but chosen to reflect the prudential degree of over-collateralisation implicit in the observed financing case. In particular, the realised pledge ratio of 27.79% implies a reciprocal coverage multiple of approximately 3.60, which provides an empirical anchor for the baseline calibration. Under this interpretation, $\upkappa$=3.6 captures the lender-relevant haircut embedded in the transaction more faithfully than the theoretical lower-bound case of $\upkappa$=1, while preserving a monetisation threshold that is consistent with the conservative structure of the observed loan. Lower values of $\upkappa$ were examined in calibration checks, but they generated threshold levels that were too low relative to the observed financing conservatism and led to near-saturated option values.

The baseline Black–Scholes inputs are therefore defined as follows. The current underlying price is ${S}_{0}=$ CNY 60 per tCO₂e. The strike price $\mathrm{X}$ is determined by the monetisation-threshold expression above. Time to maturity is set to $\mathrm{T}=3$ years, consistent with the observed loan term. The risk-free rate $\mathrm{r}$ is taken from the three-year Chinese government bond yield at the valuation date. In the baseline specification, $\mathrm{r}$=2.33%, corresponding to the three-year Chinese government bond yield on 30 September 2022. Annualised volatility $\upsigma$ is estimated from unsmoothed weekly log returns in the pre-loan sample, so that the option valuation is based on the same thin-market price record used in the forecasting exercise.

To avoid overstating option value through volatility estimates dominated by early thin-market episodes, the annualised volatility parameter is specified in two layers. The baseline volatility is estimated from the most recent 52 calendar weeks preceding loan origination, ending on 30 September 2022, using the unsmoothed observed weekly CCER price series. A high-volatility scenario is then constructed using the full pre-loan sample from 31 August 2018 to 30 September 2022. In both cases, returns are computed from adjacent observed prices without mechanical interpolation of missing weeks. Let ${P}_{i}$ denote the observed price at week $\mathrm{i}$, and let ${\Delta}_{i}$ denote the elapsed number of weeks between adjacent observed prices. The raw log return is defined as $g\_i=\mathrm{ln}\left({P}_{i}/{P}_{i-1}\right))$, and the gap-adjusted return is defined as ${z}_{i}={g}_{i}/\sqrt{{\Delta}_{i}}$. Annualised volatility is then calculated as $\upsigma =\mathrm{s}\mathrm{d}\left({z}_{i}\right)\sqrt{52}$. Because the plain recent-window estimate remains strongly influenced by extreme thin-market movements, the baseline volatility is taken from the 5% winsorised distribution of gap-adjusted weekly returns, yielding $\upsigma$=1.7028. The high-volatility scenario retains the full pre-loan estimate, for which $\upsigma$=4.1400. An alternative robust estimate based on the median absolute deviation is reported in the Supplementary Information.

Using these inputs, the per-unit option value is obtained from the Black–Scholes call formula:

32 $$\mathrm{c}={S}_{0}\mathrm{N}\left({d}_{1}\right)-\mathrm{X}{e}^{-rT}\mathrm{N}\left({d}_{2}\right)$$

where

33 $${d}_{1}=\frac{\mathrm{ln}\left({S}_{0}/X\right)+\left(r+0.5{\upsigma }^{2}\right)T}{\upsigma \sqrt{T}}$$

and

34 $${d}_{2}={d}_{1}-\upsigma \sqrt{T}$$

The present value of the project is defined as

35 $$\mathrm{P}\mathrm{V}={S}_{0}{Q}_{\mathrm{e}\mathrm{f}\mathrm{f}}$$

the total option value is

36 $$\mathrm{C}\mathrm{V}=\mathrm{c}{Q}_{\mathrm{e}\mathrm{f}\mathrm{f}}$$

and the total economic value of the pledged carbon-related asset is

37 $$\mathrm{T}\mathrm{V}=\mathrm{P}\mathrm{V}+\mathrm{C}\mathrm{V}$$

This decomposition distinguishes the immediately recognisable collateral value from the additional value associated with waiting under uncertain future market conditions.

The pledge-rate model retains the option-theoretic structure set out above, but all inputs are aligned with the observed financing contract. The borrowing rate $\mathrm{R}$ is fixed at the contractual annual rate of 3.65%, maturity is fixed at three years, the risk-free rate $\mathrm{r}$ is the corresponding three-year government bond yield, and the volatility term $\upsigma$ follows the same two-layer specification used in the valuation module. The closed-form pledge-ratio equation therefore continues to reflect the interest-rate spread, maturity, and market volatility, while deliverability risk enters the financing valuation through ${Q}_{\mathrm{e}\mathrm{f}\mathrm{f}}$, and hence through total collateral value.

Under the baseline parameterisation, with $\theta =0.90$, $\kappa =3.6$, $r=2.33$, and a 5% winsorised recent-window volatility estimate of $\sigma =1.7028$, the per-unit option value is 50.9629 CNY per t ${CO}_{2}$ e. This implies a total option value of 33,009,270.22 CNY and a total economic value of 71,871,990.22 CNY, compared with a present-value benchmark of 38,862,720.00 CNY. Under this baseline specification, the option component remains substantial, but no longer approaches the near-saturated levels generated by lower threshold settings or by volatility estimates dominated by extreme thin-market movements. Rather, it reflects the economically meaningful value of timing flexibility once the monetisation threshold is aligned with the prudential structure of the observed transaction.

Solving Eq. (27) under the same baseline parameterisation yields a pledge ratio of $w=0.0824$, corresponding to an implied pledgeable amount of 5,922,484.79 CNY. This value is lower than the observed loan amount of RMB 12 million and should not be interpreted as a literal prediction of realised bank lending. Rather, it represents a theoretical lower-bound pledge ratio consistent with non-loss lending under the stated assumptions of the model. The observed pledge ratio of 27.79% is defined on the original bank appraisal basis of RMB 43.18 million, whereas the present framework applies the pledge ratio to the model-defined total economic value $TV$. For reference, when the observed loan amount is rescaled by the model-defined denominator, the implied observed effective ratio is approximately 16.7%, which remains above the model-implied benchmark. This comparison indicates that the realised transaction embeds additional prudential buffers and institutional judgement beyond the lower-bound break-even condition identified by the model. Such conservatism may plausibly reflect internal risk limits, collateral-management practices, liquidation discounts, verification uncertainty, or supplementary haircuts not directly observable in the public case record.

To examine the sensitivity of the valuation to extreme volatility conditions, a high-volatility scenario is further evaluated using the full pre-loan estimate of $\sigma$. Under this stress specification, the per-unit option value rises to 59.9783 CNY per t ${CO}_{2}$ e, the total option value increases to 38,848,667.19 CNY, and the total economic value rises to 77,711,387.19 CNY. Relative to the baseline case, the high-volatility scenario increases total collateral value by 5,839,396.97 CNY. This result reinforces the distinction between collateral valuation and lendable value: more extreme volatility can raise the option component of collateral value, but it does not by itself imply that realised lending should expand proportionately. The stress case is therefore best interpreted as a bounded upper-risk scenario for collateral valuation rather than as an alternative benchmark for observed bank practice.

The CNN–LSTM forecasts remain relevant within this framework, but their role is distinct from that of the strike. The model’s future 4-week average forecasts are used to characterise the forward-looking market environment relevant to monetisation and to anchor the scenario design reported below. They therefore provide forward-looking market information for valuation, while the strike continues to represent the economically grounded threshold at which monetisation becomes rational. In this sense, forecasting informs the market environment surrounding monetisation, whereas the strike represents a contract-grounded debt-coverage threshold rather than a mechanically predicted future price.

### Sensitivity analysis of pledge financing valuation under varying parameter scenarios

To examine how the financing capacity of forest-based carbon collateral responds to uncertainty, the sensitivity analysis considers three lender-relevant dimensions—CCER market price, effective deliverability, and volatility identification—together with a calibration check for the prudential coverage factor $\upkappa$. For each scenario, the model recalculates the monetisation threshold $\mathrm{X}$, the per-unit option value $\mathrm{c}$, present value $\mathrm{P}\mathrm{V}$, option value $\mathrm{C}\mathrm{V}$, total economic value $\mathrm{T}\mathrm{V}$, pledge ratio $\mathrm{w}$, and the implied pledgeable amount ${L}^{*}$.

**Fig. 3:**
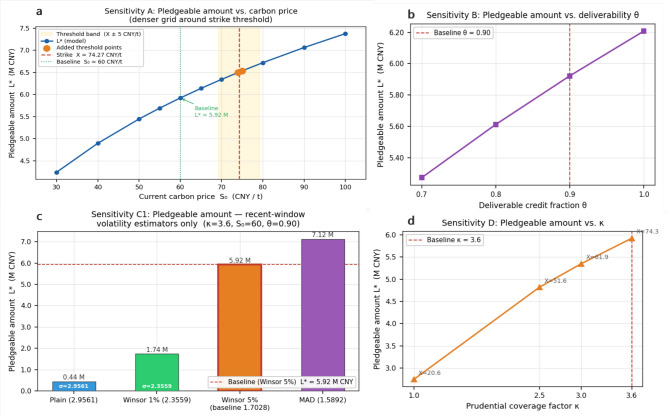
Sensitivity of the model-implied pledgeable amount to price, deliverability, volatility estimation and threshold calibration. (**a**) Model-implied pledgeable amount ${L}^{*}$ under alternative CCER prices ${S}_{0}$. Vertical lines indicate the baseline price and baseline strike. (**b**) ${L}^{*}$ under alternative deliverable credit fractions $\theta$, where ${Q}_{eff}=\theta {Q}_{nom}$. (**c**) ${L}^{*}$ under alternative recent-window volatility estimators, with the 5% winsorised estimate used as the baseline specification. (**d**) Calibration check for the prudential coverage factor $k$, with annotations indicating the implied strike values. In all panels,${L}^{*}=w\times TV.$.

Figure 3a shows the sensitivity of the model-implied pledgeable amount to the current CCER price. The relationship is strongly positive: higher prices increase the immediately recognisable collateral value and, for a given threshold setting, also strengthen the option component embedded in delayed monetisation. The response is not purely linear, however. The most policy-relevant variation occurs in the vicinity of the monetisation threshold, where relatively small changes in price translate into more pronounced changes in pledgeable capacity. Once prices move sufficiently above the threshold, the financing response remains positive but becomes less steep. This pattern reflects the combined non-linearity of the option-pricing structure and the pledge-ratio equation.

Figure 3b examines sensitivity to effective deliverability through the deliverable credit fraction $\uptheta$, where ${Q}_{eff}=\uptheta {Q}_{nom}$. A higher $\uptheta$ increases pledgeable capacity through two linked channels. First, it directly enlarges the collateral base entering both $\mathrm{P}\mathrm{V}$ and $\mathrm{C}\mathrm{V}$. Second, because the unit monetisation threshold is inversely related to ${Q}_{eff}$, greater effective deliverability lowers the price required to justify monetisation and thereby strengthens the option component. Conversely, lower deliverability compresses collateral value and tightens the financing constraint. This result highlights that, for forest carbon assets, lender-relevant quantity is not simply nominal project volume but the proportion of that volume that can prudently be treated as effectively deliverable collateral.

Figure 3c compares the model-implied pledgeable amount under alternative recent-window volatility estimators. The results show that volatility identification is not a secondary technical choice, but a material determinant of financing outcomes. The plain gap-adjusted estimator yields the lowest pledgeable amount, whereas the median-absolute-deviation-based estimate yields the highest among the recent-window specifications. The 5% winsorised estimate, adopted as the baseline, lies between these bounds and provides a conservative yet economically interpretable recent-window measure. The comparison therefore supports the use of a robust volatility estimator in thin-market settings, where untreated return extremes can otherwise compress the pledge-ratio channel mechanically.

Figure 3d reports a calibration check for the prudential coverage factor $\upkappa$. Unlike the preceding panels, this is not intended as a market-risk sensitivity in the narrow sense, but as a test of how alternative threshold settings affect financing outcomes under the same contract structure. Across the tested range, higher values of $\upkappa$ imply higher strike levels and more conservative monetisation thresholds. In the present calibration, the resulting changes in the pledge-ratio channel dominate the associated reduction in total collateral value, so that the model-implied pledgeable amount increases across the tested range. This confirms that $\upkappa$ is a consequential structural parameter and that its baseline setting should be treated as an explicit calibration choice rather than as a purely mechanical input.

Taken together, the results show that pledge-based financing capacity is shaped jointly by market conditions, effective deliverability, volatility identification and prudential threshold calibration. The central implication is that the financing value of forest-based carbon assets cannot be inferred from nominal project volume or spot-market prices alone, nor can higher economic collateral value be assumed to translate mechanically into a larger lendable amount. A lender-relevant valuation framework must instead distinguish between economic asset value and model-implied lending capacity, and integrate price risk, implementation risk and calibration discipline within a common collateral-pricing system.

## Discussion

### Key research findings and insights

This study develops a contract-consistent framework for pricing forest-based carbon collateral under uncertainty, and its central finding is that economic collateral value and lendable value are not the same object. The distinction matters because much of the existing discussion around forest-carbon finance implicitly assumes that better asset valuation should translate directly into greater lending capacity. The present results show that this assumption is too simple. Under a contract-consistent treatment of maturity, repayment structure, deliverability risk, volatility identification and prudential threshold calibration, option-adjusted collateral value can increase materially while the model-implied lendable amount remains tightly compressed.

The conceptual contribution of the paper is therefore to identify a structural wedge between collateral revaluation and lending capacity in forest-based carbon pledge finance. The methodological contribution is to show how this wedge can be analysed within a single workflow that links forward-looking market information, threshold-based option valuation, effective deliverability haircuts and lender-oriented pledge-rate determination. Recent evidence that existing forest offset buffer pools may be materially undercapitalized against disturbance-driven carbon losses further suggests that permanence risk cannot be assumed to be fully neutralised by current offset-market design^12^. Rather than treating forecasting, valuation and pledge-rate choice as separate exercises, the framework integrates them into one pricing system anchored to the observed financing contract. The empirical contribution is to demonstrate, in the Ning’er case, that a higher option-adjusted collateral value does not automatically imply that banks should lend more. Instead, once downside-protection logic is imposed, the model-implied pledgeable amount is better interpreted as a lower-bound break-even benchmark than as a literal prediction of observed bank practice.

This re-framing sharpens the economic relevance of the paper. The study is not simply about improving the valuation of a new asset class. It is about showing why natural-capital collateral cannot be translated into credit allocation by asset pricing alone. For climate finance, this means that the bottleneck is not only whether forest-carbon assets can be valued more rigorously, but also how much of that value can credibly survive the transition from market-facing asset valuation to lender-facing collateral recognition.

The Ning’er case illustrates this distinction in concrete terms. First, the results confirm that valuation of forest-based carbon collateral must incorporate uncertainty and timing flexibility; a static present-value benchmark alone is insufficient in a thin, segmented and policy-sensitive market. Second, the case shows that option-adjusted collateral revaluation and lender-relevant financing capacity can diverge materially. In the baseline specification, total economic collateral value rises, yet the model-implied pledge ratio and pledgeable amount remain below the realised transaction once prudential break-even logic is imposed. Third, the case demonstrates that implementation choices often treated as technical details, including deliverability assumptions, volatility identification and threshold calibration, are in fact economically decisive because they govern how much of the revalued asset base survives into lendable collateral. The broader implication is that climate-aligned collateral pricing for natural-capital assets must be analysed as a constrained translation problem, not as a simple extension of asset valuation.

## Conclusion

This study develops and empirically applies a contract-consistent framework for forest-based carbon pledge lending under uncertainty. Its main contribution is to show that the economic value of forest-carbon collateral and its lender-relevant lendable value are analytically distinct and can diverge sharply once implementation risk, volatility identification, threshold calibration and prudential lending logic are incorporated. In the Ning’er case, option-adjusted valuation raises measured collateral value, but the model-implied pledgeable amount remains materially below observed lending practice when interpreted through a lower-bound non-loss benchmark. The paper therefore shifts the discussion from whether forest-carbon assets can be valued more highly to how much of that value can credibly be recognised as lendable collateral.

More broadly, the findings suggest that the key challenge in financing nature-based carbon assets is not only one of better measurement or better forecasting, but of translating revalued natural capital into bankable collateral under conservative institutional constraints. By linking forecasting, option-based valuation, deliverability haircuts and lender-oriented pledge-rate determination in a single contract-consistent framework, the study provides an empirical basis for analysing this translation problem. The Ning’er case should be read as an institutional and methodological illustration rather than as proof of universal operational readiness. Even so, the framework clarifies a point of wider relevance for climate finance: rigorous asset valuation is necessary, but not sufficient, for unlocking credit against natural-capital collateral.

### Policy recommendations

The policy implications of this study should not be read as implying that higher modelled collateral value ought automatically to translate into more aggressive lending. Rather, the results point to the need for greater transparency, comparability and risk sensitivity in the translation of forest-carbon asset value into lender-recognised collateral. Based on the findings of this study, several targeted policy recommendations are proposed to promote the healthy development of the forest-based carbon asset market and to improve the financing infrastructure associated with pledge-based mechanisms.

First, the government should accelerate the streamlining and standardisation of the CCER project approval process. Since 2017, the National Development and Reform Commission suspended approvals of CCER projects due to various concerns, including low market enthusiasm, inconsistencies in project quality, and excess supply. On 22 January 2024, China officially relaunched its national voluntary greenhouse gas emissions trading market. With this relaunch came the establishment of a new and more rigorous regulatory framework for project registration. However, to sustain market confidence and efficiency, further procedural refinement is required. By improving transparency, maintaining quality standards, and stabilising the supply of CCERs, the government can energise the voluntary carbon market and attract more corporate actors to engage in forestry carbon trading.

Second, commercial banks must develop formalised evaluation standards for carbon asset pledging. Currently, pledge-based financing mechanisms for forestry carbon assets lack consistent institutional criteria. Banks should implement structured assessment frameworks that incorporate firms’ emissions performance, adoption of decarbonisation technologies, and governmental incentive or penalty schemes. Such standardisation would enhance banks’ capacity to identify and support viable carbon asset pledge applications while mitigating the risks of asset mispricing or undercollateralisation.

Third, project owners must strengthen the internal management of forestry carbon sink initiatives. This includes building robust internal training systems to ensure professional literacy across departments, and establishing dedicated teams for coordination with verification bodies and regulatory agencies. By leveraging third-party expertise in project assessment and monitoring, enterprises can accelerate approval timelines and reduce compliance risks. Furthermore, sustained attention should be given to post-registration project management, including the development of transparent and methodologically sound monitoring systems to enable accurate calculation of carbon sequestration volumes. Such systems not only inform carbon trading strategies but also improve investor confidence. Ultimately, by improving internal governance, enterprises can enhance both their financial performance and their contribution to national emissions reduction targets.

Looking ahead, several promising directions for future work may be pursued.

A key avenue for future research lies in extending the framework beyond single-case collateral valuation towards richer modelling of the wedge between asset value and lendable value. This includes not only improvements in carbon price forecasting—such as multi-factor models incorporating policy, energy and macroeconomic variables—but also better empirical identification of deliverability risk, liquidation conditions, and institution-specific threshold calibration. Advancing these components jointly will be more important than improving forecasting accuracy in isolation, because the main challenge for carbon-based natural capital lies in the prudential translation of value into credit.

Moreover, as carbon markets mature and become more integrated with other financial and commodity markets, research on cross-market linkages and risk transmission mechanisms will become increasingly salient^49^. Exploring the interdependence between carbon, energy, and financial markets—as well as between national and international carbon trading systems—can help to clarify how shocks propagate across platforms. Constructing a comprehensive analytical framework for cross-market carbon dynamics would provide scientific support for policy formulation and risk management.

Additionally, the adoption of emerging technologies such as blockchain can substantially improve market transparency and transaction efficiency. Applying blockchain to carbon markets could enable the development of secure, decentralised, and verifiable transaction systems, thereby increasing the integrity and scalability of carbon asset pledging. This would be especially relevant for forestry carbon projects where measurement, reporting, and verification are often complex and fragmented.

Finally, there is great potential in expanding the range of financial instruments linked to forestry carbon assets. Combining forestry carbon credits with derivatives such as carbon futures and carbon options would diversify the ecosystem of green financial products and offer firms new tools for risk management and credit access. In this way, forestry carbon finance could move beyond simple collateralisation and towards more sophisticated asset-backed solutions.

Taken together, these directions offer a roadmap for deepening the intersection of forestry, climate finance, and technological innovation, while reinforcing the credibility, functionality, and sustainability of carbon pledge financing in China and beyond.

## Supplementary Information

Supplementary Information.

## Data Availability

The weekly transaction data underlying this study, together with the model-ready datasets and main result tables generated during the revision process, have been deposited in Figshare 10.6084/m9.figshare.32055291.
