# Supplementary material for: Unlocking the financing potential of forest-based carbon assets: a valuation framework for pledge lending under uncertainty in China
Source: Sci Rep. 2026 May 18;16:19558. doi: 10.1038/s41598-026-53268-y (PMC13294374; doi:10.1038/s41598-026-53268-y)
Supplement: Supplementary file 1 — Supplementary Information. [file 41598_2026_53268_MOESM1_ESM.docx]

Supplementary Information

Supplementary Information for: Unlocking the Financing Potential of Forest-Based Carbon Assets: A Valuation Framework for Pledge Lending under Uncertainty in China

1. Background: Forest-Based Carbon Asset Collateralisation in China

Forest-based carbon assets represent the market-oriented transformation of ecosystem services, and their developmental trajectory has closely followed the evolution of China’s carbon trading frameworks. In the early stages, such projects were primarily linked to international mechanisms, notably under the Clean Development Mechanism (CDM). China emerged as a major host country for forestry CDM initiatives. However, the high technical thresholds, extended verification cycles, and increasing volatility of international carbon markets gradually weakened this pathway. Since 2011, with the institutionalisation of pilot carbon markets and the introduction of China Certified Emission Reductions (CCER), forest-based carbon assets have transitioned from internationally driven projects to domestically integrated components of national climate policy.

Currently, these assets operate under a dual-track system: nationally approved methodologies for afforestation, forest management, and bamboo carbon projects provide the accounting standards, while regional pilot programmes implement differentiated offset mechanisms. Forest-based carbon assets now span national CCER projects, subnational initiatives (e.g., in Fujian, Guangdong, Guizhou), and localised schemes involving individual smallholder participation. As a result, they constitute one of the most diversified and multilayered ecological asset classes within China’s carbon market landscape.

Despite this institutional expansion, forest-based carbon assets face persistent challenges related to liquidity and financial viability. Long project cycles, high initial costs, and delayed revenue generation limit their attractiveness as conventional loan collateral. Simultaneously, constrained CCER supply and delays in new issuance have led to tight market conditions, with carbon credit prices increasingly driven by policy expectations and demand shocks, further amplifying valuation uncertainty.

In response, pledge-based financing has emerged as a promising mechanism to monetise forest-based carbon assets. Two dominant structures are now in use: (1) pledging verified emission reductions as collateral for credit access, which benefits from clear asset certification and relatively stable creditworthiness; and (2) pledging future carbon revenue rights, which expands financing potential but imposes higher requirements for price forecasting, verification credibility, and default management. Complementary instruments such as carbon ticket pledges and hybrid structures combining carbon revenues with industrial earnings have also been piloted in local markets to address liquidity constraints and bridge the valuation gap.

Taken together, the development of forest-based carbon assets in China has shifted from an ecological conservation logic to an increasingly market-based model. However, their integration into financial systems remains hindered by three critical mismatches: first, the temporal misalignment between long carbon cycles and short financial tenures; second, the informational asymmetry between ecological value and financial risk pricing capacity; and third, the structural misfit between project cash flow characteristics and standardised financial products. To enable forest-based carbon assets to serve as an effective bridge between carbon markets and green finance, future efforts must address institutional bottlenecks in property rights clarification, enhance technical capacity in asset valuation, and expand the range of structured financing tools capable of accommodating the unique attributes of carbon-based natural capital.

The Development Trajectory of Forest-Based Carbon Assets in China

The concept of forest-based carbon assets was formally institutionalised under the Kyoto Protocol, which recognised afforestation and reforestation as legitimate tools for mitigating climate change. These projects were initially implemented under the Clean Development Mechanism (CDM), where forest-related interventions—including tree planting and forest ecosystem management—were designed to capture atmospheric carbon dioxide and convert it into tradeable environmental assets. Such integration allowed for the simultaneous realisation of ecological and financial objectives.

China’s engagement with forest-based carbon asset development began in 2006, when the world’s first CDM forestry project was launched in the Pearl River Basin of Guangxi Province. This was soon followed by a range of initiatives in carbon-rich regions such as Inner Mongolia. After 2007, the expansion of CDM forest projects accelerated, supported by international climate finance and technical cooperation. However, by 2013, growth had slowed considerably. Challenges in institutional regulation, project verification, and governance coherence hindered progress, leading to a downturn in the overall development of forest-based carbon asset initiatives.

In 2011, China began piloting emissions trading systems (ETS) in several provinces and municipalities. By 2012, the Chinese Certified Emission Reduction (CCER) scheme was formally incorporated into the domestic trading architecture, serving as a voluntary offset mechanism. In this context, forest-based carbon assets were explicitly defined as an eligible project type for CCER registration. To date, nine provincial-level ETS pilots have embedded CCER credits into their compliance offset mechanisms, marking a strategic shift in China’s engagement with carbon markets—from an internationally driven system to a domestic, policy-led one.

Methodologically, four categories of forest-based carbon asset projects have been approved for CCER development: afforestation, forest management, bamboo forest carbon assets, and bamboo operational schemes.

In terms of participation scale, China’s forest-based carbon assets are now embedded within international, national, and sub-national regimes. At the international level, projects align with CDM, the Voluntary Carbon Standard (VCS), and the Gold Standard (GS). At the national level, they are registered under the CCER framework. At the regional level, projects are linked to schemes such as the Beijing Forestry Offset Programme, the Fujian Provincial CCER System, the Guangdong Inclusive Carbon Mechanism, and the Guizhou Single-Tree Carbon Asset Initiative.

As shown in Supplementary Figure 1, international participation has primarily adhered to CDM, VCS, and GS methodologies. According to publicly available records, China has developed a total of 38 forest-based carbon asset projects under these frameworks. Domestically, 97 projects have been approved under the CCER methodology.

Regional statistics illustrate a growing institutional diversity and trading volume. As of May 2024, Fujian Province had completed trades totalling 4.1263 million tonnes of verified forest-based carbon assets, amounting to CNY 64.77 million in transaction value. As of April 2023, Guangdong Province recorded 5.0248 million tonnes of traded voluntary emission reductions worth CNY 115 million. In Guizhou, the Single-Tree Carbon Asset Project involved over 10,000 participating forest households, with cumulative transactions reaching CNY 13.47 million.

These developments reflect the layered evolution of China’s forest-based carbon asset governance—from international project-based mechanisms to nationally integrated systems, and further to localised pilot innovations. They also signal significant room for market consolidation, standardisation, and future expansion.


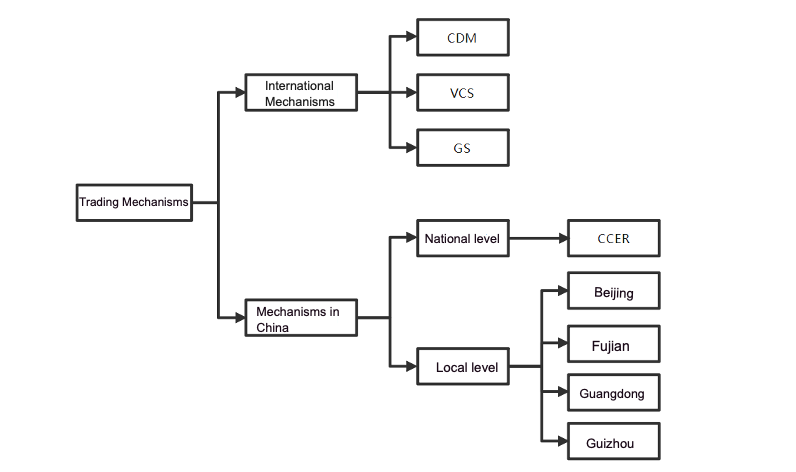


Supplementary Figure 1. | Institutional structure of forest-based carbon asset trading mechanisms in China and related international frameworks.

CCER Project Development Process and Trading Status

Within China’s emissions trading scheme (ETS) pilots and the national carbon market, the **Chinese Certified Emission Reduction (CCER)** mechanism functions as a core voluntary offset instrument. Forest-based carbon asset projects developed under this mechanism follow a standardised three-stage process. The associated documentation, in sequential order, includes: (1) a **Feasibility Assessment Report**, (2) a **Project Design Document (PDD)** for registration, and (3) a **Project Validation and Verification Report**.

China’s CCER market has also undergone a pronounced institutional discontinuity that is highly relevant to the valuation of forest-based carbon assets. Following the suspension of new CCER project registration in March 2017, the market remained closed to new project approvals for nearly seven years, with only previously registered legacy projects continuing to circulate. During 2023, the Ministry of Ecology and Environment accelerated the reconstruction of the institutional framework for the national voluntary carbon market, culminating in the release of the Administrative Measures for Voluntary Greenhouse Gas Emission Reduction Trading (Trial) on 20 October 2023. The national CCER market was formally relaunched on 22 January 2024. Under the transitional arrangements, only CCERs registered before 14 March 2017 remained eligible for offsetting compliance obligations until 31 December 2024, and such use ceased from 1 January 2025 onwards. This institutional break is important for the present study because the observed loan was issued in September 2022 and therefore spans both the prolonged suspension phase and the initial stage of market restart. Accordingly, CCER price and liquidity conditions over the contract period cannot be treated as if they arose from a fully continuous and homogeneous market environment.

The purpose of the feasibility assessment is to determine whether a project satisfies the baseline eligibility criteria and conforms to the approved CCER methodologies. Professional evaluators are engaged to review the proposed intervention, ensuring alignment with sectoral and technical guidelines. Under the updated CCER regulatory framework, project developers submit applications directly to the Ministry of Ecology and Environment, which centrally oversees approval procedures. Once approved, all CCER units are issued and traded via a centralised platform operated by the Beijing Green Exchange.

The preparation of the PDD is a critical step in the project development cycle. This document must comprehensively detail the project’s background, technical plan, baseline methodology, and anticipated emissions reductions. It also provides justification for additionality, demonstrating that the emissions reduction would not have occurred under a business-as-usual scenario. Upon submission, the project undergoes a validation review by an accredited third-party body. If all criteria are met, the project is formally registered with the authorities.

In the final stage, the project owner engages an authorised verification body to conduct post-implementation monitoring and carbon quantification. Based on a review of documentation and site-specific evidence, the verifier issues a **Verification Statement**, which is then submitted to the National Development and Reform Commission (NDRC) or its successor for confirmation. Once approved, the certified emissions reductions are registered in the **National Voluntary Emission Reduction Registry**, enabling formal entry into the trading system.

The end-to-end development process typically takes a minimum of **five months** to complete. Each procedural step requires a high degree of technical rigour and institutional coordination, resulting in relatively long project cycles and a strong emphasis on compliance.

At present, **nine national CCER trading platforms** operate across China. In **2024**, the total annual transaction volume of CCERs across these platforms reached approximately **20 million tonnes**, representing a **29% year-on-year increase**. However, due to a multiyear pause in the issuance of new CCER credits, the market experienced a notable supply–demand imbalance. As a result, CCER prices steadily increased, reaching **CNY 100 per tonne** in the fourth quarter of 2024, approaching the price level of compliance market allowances (CEA).

Two primary factors explain this price escalation. First, the **available stock of CCER credits** has become increasingly constrained. While trading volume has surged, the absence of newly issued credits in recent years has reduced overall liquidity, creating a structural shortfall. Second, the **relaunch of the national voluntary carbon market in January 2024**, coupled with **enhanced regulatory oversight** of carbon credit quality and transaction integrity, has led to renewed investor confidence. This, in turn, has stimulated increased engagement with forest-based carbon asset projects, elevating both trading frequency and market prices.

These dynamics indicate strong growth potential for the CCER mechanism moving forward. Supported by evolving policy incentives, rising corporate climate ambitions, advances in monitoring technology, and China's broader efforts to internationalise its carbon finance architecture, CCERs are poised to play an increasingly influential role in the country’s strategy for addressing climate change and facilitating low-carbon development.

Coevolution Between Forest-Based Carbon Assets and Carbon Market Development

As the institutional platform for emissions trading, the carbon market plays a pivotal role in determining the operational efficiency and allocative effectiveness of emissions rights mechanisms. The recent **relaunch of the CCER scheme** has revitalised China’s carbon market, reintroducing momentum to both compliance and voluntary sectors. The integration of **forest-based carbon assets** into carbon market operations has created a dynamic mechanism through which such assets can contribute to price stabilisation and liquidity enhancement.

Once forest-based carbon assets are embedded within the economic logic of carbon markets, they provide enterprises with expanded avenues for offsetting emissions obligations. Their entry significantly enriches the array of tradable instruments, enhancing both the flexibility and diversity of market operations. First, by offering investors a broader selection of carbon-linked financial products, forest-based carbon assets enable **more flexible risk management strategies**. This product innovation not only reduces overall market volatility but also shifts investor attention toward environmental performance metrics.

Second, the implementation of forestry carbon projects improves **investment efficiency** for market participants. Under capital-constrained conditions, investors may monetise carbon credit entitlements derived from forestry projects to generate returns at relatively low marginal cost. This promotes **efficient resource allocation**, whereby capital is directed toward projects with both ecological and financial yield.

Third, the stabilising influence of forest-based carbon assets is also manifested in their contribution to **market expectations**. These projects typically provide **predictable cash flows** and **relatively stable revenue forecasts**, thereby fostering investor confidence in the long-term viability of green assets. This can steer trading activities towards more sustainable and fundamentally supported behaviour, encouraging the emergence of a mature and resilient market ecosystem.

As the integration of forestry, wind energy, and other carbon credit-generating projects deepens across China’s carbon market architecture, the market’s **macro-regulatory and allocative capacities** are increasingly evident. This consolidation of instruments supports more precise emissions rights management and facilitates the realisation of national climate goals.

Within this framework, forest-based carbon assets have emerged as an important class of **green financial products**, particularly in the domain of **pledge-based financing**. This is evident in several key respects:

First, a range of **pledge financing models** has emerged. The most common include pledging of **verified emissions reductions (VERs)** and **forward-looking credit entitlements**. In the former, investors use certified carbon reductions as collateral, derived from actual forestry operations. In the latter, the future value of expected carbon outputs is pledged—typically backed by pre-registration estimates and verified methodologies. Additional mechanisms such as **carbon invoice pledging** and **dual-credit models** (linking carbon revenues with industrial income streams) are also gaining traction.

Second, the use of **carbon rights as collateral** significantly **lowers financing costs**. By transforming carbon resources into recognised financial assets, investors reduce their dependency on conventional debt instruments, thus decreasing interest burdens and increasing access to capital.

Third, this pledge-based model fosters **green financial innovation**. The success of forestry carbon pledging has provided a blueprint for other carbon-intensive sectors—such as energy and transport—to pursue similar financing arrangements. In this way, pledge-based mechanisms can be leveraged to alleviate sector-specific credit constraints and catalyse low-carbon transitions.

Fourth, **green investment channels have expanded** as a result. Carbon asset pledge financing not only delivers direct exposure to carbon revenues, but also enables investors to participate—indirectly but meaningfully—in broader environmental governance. This dual function, combining economic return with environmental contribution, offers a compelling new route for aligning capital markets with sustainability objectives.

In sum, the deeper incorporation of forest-based carbon assets into China's carbon trading infrastructure has enhanced market liquidity, diversified financial instruments, and strengthened the alignment between ecological objectives and financial innovation. As such, these developments position forestry-based instruments as a strategic component of China’s evolving green finance architecture.

2. Analytical background and study-specific implementation of the B-S and CNN–LSTM framework

Study-specific empirical implementation of the forecasting and valuation framework

The empirical implementation used in this study follows the contract-consistent design set out in the main text. The observed loan is treated as originating on 30 September 2022, with a contractual maturity of three years, a fixed annual borrowing rate of 3.65%, and a bullet repayment structure under which principal and accrued interest are settled in full at maturity. The valuation exercise is therefore aligned with the observed financing structure rather than with a simplified one-year benchmark.

The forecasting component is based on unsmoothed weekly CCER transaction data rather than on monthly observations. The pre-loan sample spans 31 August 2018 to 30 September 2022, and the forecasting target is defined as the future 4-week average transaction price. This design is intended to recover a forward-looking price environment relevant to collateral valuation under thin-market conditions, rather than to maximise high-frequency predictive fit.

In the valuation module, the strike is not defined as a point forecast generated by the neural network. Instead, it is specified as a contract-grounded monetisation threshold derived from the observed financing structure. The effective collateral quantity is defined as $Q_{eff}=\theta Q_{nom}$, where $\theta$ captures prudential reductions associated with MRV uncertainty, verification delay, permanence risk, reversal risk, and related implementation frictions. The repayment obligation at maturity is defined under an annual compounding convention as $D_{T}=L_{0}(1+R)^{T}$, and the strike is specified as $X=\kappa D_{T}/Q_{eff}$.

The volatility input is likewise implemented in a study-specific way. Baseline volatility is estimated from recent-window weekly gap-adjusted returns using a robust estimator, while the full pre-loan estimate is retained separately as a high-volatility stress case. This treatment is adopted to avoid allowing early thin-market episodes to dominate the baseline option value mechanically, while preserving a stress-test benchmark for extreme volatility conditions.

Black–Scholes Option Pricing Model

The assumptions listed below are retained as analytical benchmark conditions underlying the Black–Scholes pricing framework. They are not intended as literal descriptions of observed CCER market microstructure, which in the empirical analysis is thin, irregular and institutionally segmented. Options confer upon the holder the right—but not the obligation—to buy or sell an underlying asset at a predetermined price within a specified timeframe. This discretionary feature distinguishes options from traditional financial instruments. Options are categorised into European and American types, differentiated by the timing of exercise rights: European options permit exercise solely at maturity, whereas American options allow exercise at any point up to expiration. This flexibility offers investors enhanced control over their positions. A defining characteristic of options is the asymmetry between rights and obligations: the buyer possesses the right to exercise the option, while the seller bears the potential liabilities. Such mechanisms are prevalent not only in financial markets but also across various economic activities.

The Black–Scholes (B–S) model stands as a seminal framework in financial economics, providing a method for the precise valuation of options. Developed by Fischer Black and Myron Scholes in 1973, the model is predicated on a set of assumptions that facilitate the construction of an effective hedging strategy. This strategy involves dynamically adjusting a portfolio comprising the underlying asset and a risk-free bond to mitigate the risks associated with price volatility. The foundational assumptions of the B–S model include:

- The option is of the European type, exercisable only at expiration.
- The price of the underlying asset follows a geometric Brownian motion, implying that its returns are log-normally distributed.
- The underlying asset is divisible and can be sold short.
- No dividends are paid on the underlying asset during the option's life.
- Markets are frictionless, with no transaction costs or taxes.
- The risk-free interest rate is constant and known.
- There are no arbitrage opportunities.
- Trading of the underlying asset is continuous.

Under these assumptions, the B–S model derives the following formulas for pricing European call and put options:

**Call Option:**


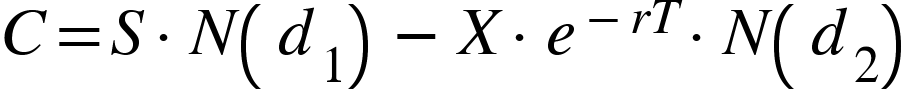


**Put Option:**


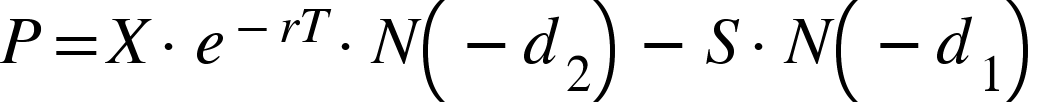


Where:


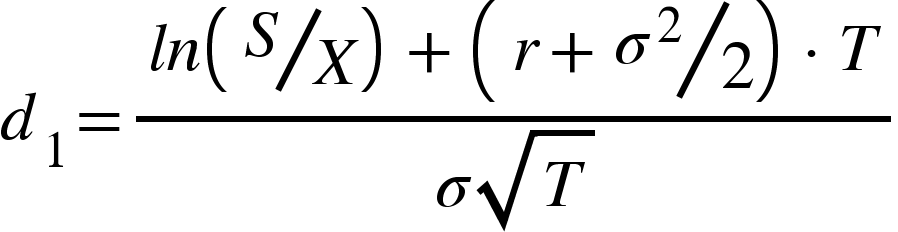


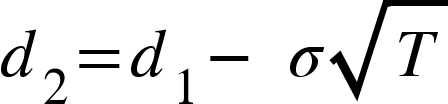


Variables are defined as follows:

$C$: Value of the call option

$P$: Value of the put option

$S$: Current price of the underlying asset

$X$: Strike price of the option

$T$: Time to expiration

$N\left( d \right)$: Cumulative distribution function of the standard normal distribution

$r$: Risk-free interest rate

$\sigma$: Volatility of the underlying asset's returns

This model has profoundly influenced the valuation of derivative securities and remains integral to contemporary financial analysis.

CNN–LSTM Model

The CNN–LSTM model is a deep learning architecture that combines a Convolutional Neural Network (CNN) with a Long Short-Term Memory (LSTM) network. It is primarily employed to handle data exhibiting complex temporal and spatial characteristics, finding widespread application in the processing of sequential data—such as in speech recognition and time series forecasting. When applied to forecasting the price of forest-based carbon assets within afforestation projects, the CNN–LSTM model effectively captures both spatial and temporal features present in the data. In this composite architecture, the CNN component processes the time-series input via convolution to extract spatially localised features, while the LSTM component extracts nonlinear temporal dependencies between successive observations. The two are then combined to predict future carbon price trends. In the following subsections, the individual CNN and LSTM components of the model are described in turn.

**Recurrent Neural Network (RNN) Foundation**

The LSTM network is an improved variant of the traditional Recurrent Neural Network (RNN). A standard RNN is a cyclic structure that connects information across different time steps, first proposed in 1982 by John Hopfield. Its topology is illustrated in Supplementary Figure 2 and provided the theoretical underpinning for the subsequent development of LSTM.


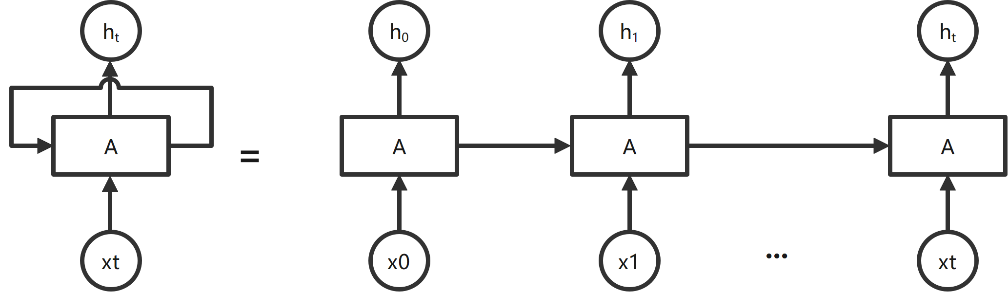


Supplementary Figure 2. | Recurrent neural network (RNN) architecture.

At time step $t$, the hidden unit $h_{t}$ receives the prior hidden state $h_{t-1}$together with the current input $x_{t}$, and computes the output for that time. Simultaneously, the previous input $x_{t-1}$ influences the current output via the network’s recurrent connections. Forward propagation proceeds in sequence order, after which back-propagation through time recomputes stored values for parameter updates—a standard training algorithm for RNNs. The forward-propagation equations for this RNN are:


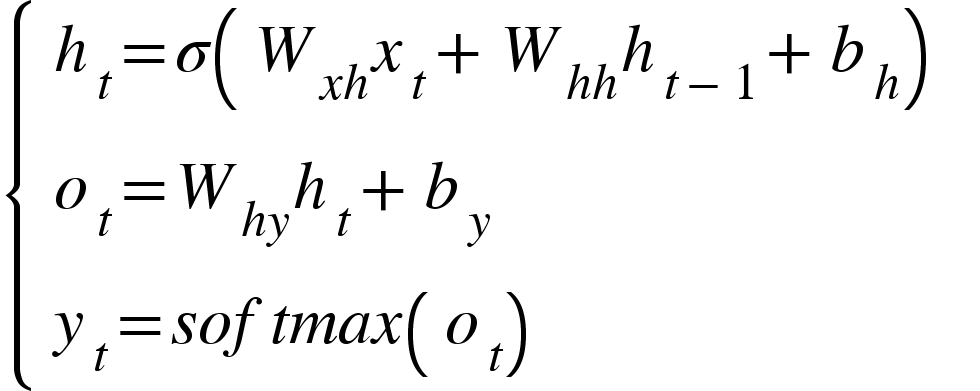


Here, $b_{y}$and $b_{h}$are bias vectors; $W_{hy}$is the weight matrix connecting the hidden unit $h_{t}$to the output, $W_{xh}$ connects the input$x_{t}$to the hidden unit, and $W_{hh}$connects the previous hidden state to the current one. Owing to parameter sharing across time steps, the RNN can in principle process sequences of arbitrary length. Because each hidden state depends on all prior hidden states, the network can model temporal dependencies over long horizons. Notably, the length of the output sequence need not match that of the input; rather, it may be flexibly chosen to suit the task.

However, as RNN depth increases, the product of Jacobian matrices can grow or decay exponentially, causing the well-known problems of vanishing or exploding gradients. To address this, back-propagation through time is used, minimising a loss function so that predicted values converge towards actual observations. Let the recurrence be defined by


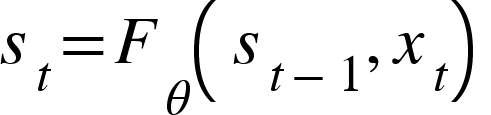


then the gradient of the loss at time$T$with respect to parameters $\theta$is


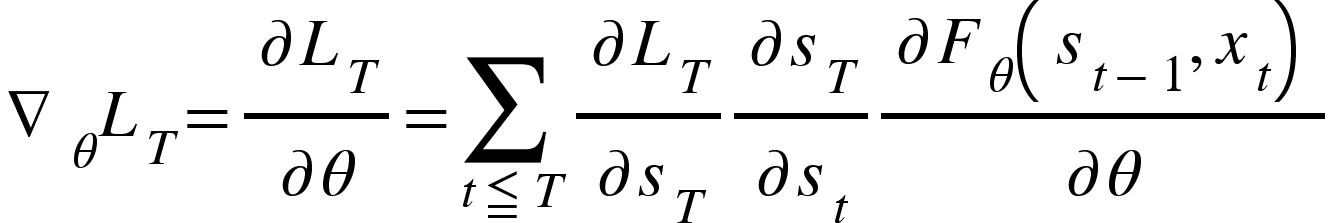


By applying the chain rule, the Jacobian $\frac{\partial s_{T}}{\partial s_{t}}$ decomposes as


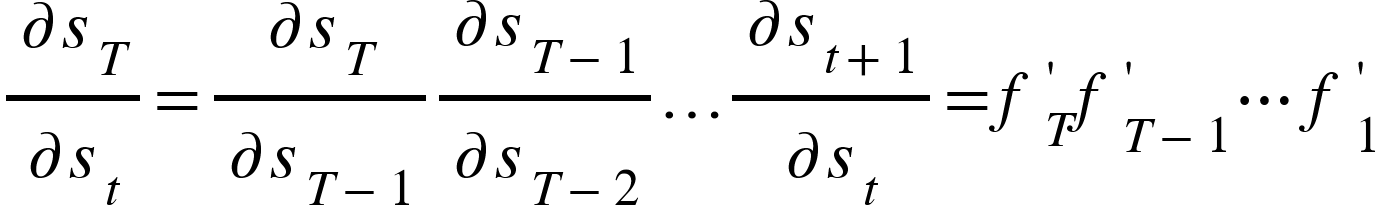


For stable information retention, one requires$\mid f_{1}'\mid<1$, yet as $T$grows the gradient $\nabla_{\theta}L_{T}$may exponentially approach zero—resulting in vanishing gradients. Conversely, if $\left| f_{1}^{'} \right|$＞1, gradients may explode. These difficulties prompted the development of more advanced recurrent cells, of which the LSTM is the foremost example.

**In recent years, the LSTM model, developed by Sepp Hochreiter and his team, has been extensively adopted in practical recurrent network frameworks. This enhanced version of the RNN outperforms the standard RNN in scenarios involving long-range dependencies, primarily because it effectively overcomes the vanishing-gradient problem that can occur during back-propagation. Although the LSTM architecture superficially resembles that of a traditional RNN, its core innovation lies in the introduction of more refined internal processing units. This improvement enables LSTM networks to manage and update information more efficiently, thereby capturing complex sequence patterns with greater fidelity. Here, we describe the standard LSTM model.**

**The LSTM achieves precise control over information flow by incorporating three gating mechanisms—an input gate, a forget gate, and an output gate—as illustrated in Supplementary Figure 3. Each gating unit employs the sigmoid function as its activation and uses element-wise multiplication to determine the direction and extent of information passage. Mathematically, each gate is expressed as:**


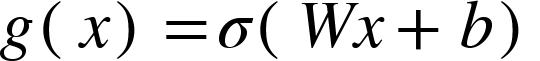


where


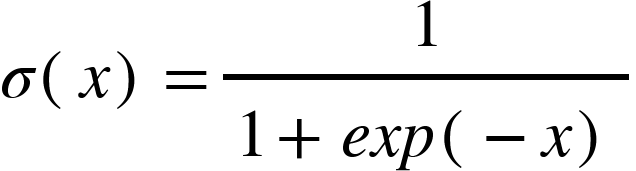


is the sigmoid function, a commonly used nonlinear activation in deep learning. Its output lies in the range [0, 1], indicating the proportion of information permitted to pass: an output of 0 denotes no passage, while an output of 1 denotes full passage.

In this model, $i$, $f$, and $o$ denote the input gate, forget gate, and output gate respectively; $\odot$ represents element-wise multiplication; and $W$ and $b$ are the weight matrices and bias vectors of the model.


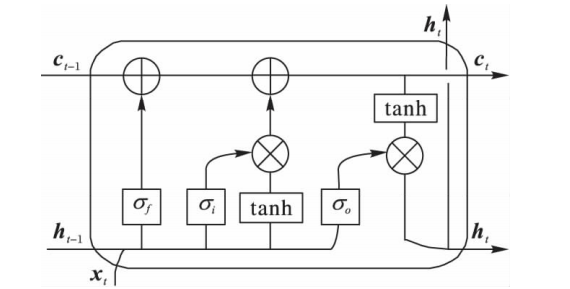


Supplementary Figure 3. | Long short-term memory (LSTM) unit structure.

The forward computations of this model are given by Equations below. At time step $t$the model inputs $x_{t}$ into the hidden layer and obtains the output sequence $h_{t}$; here, $c_{t}$denotes the cell state (memory unit). The input gate regulates how much of the input sequence $x_{t}$ is stored in the memory unit, and is defined as:


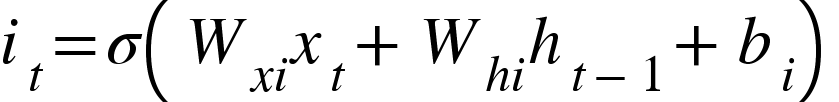


The forget gate is a crucial component of the model; by selectively retaining or discarding information, it mitigates the vanishing- or exploding-gradient issues that can arise during back-propagation through time. Specifically, the forget gate controls the activity of the recurrent connection and thus decides which portion of the previous cell state $c_{t-1}$ is transferred to the current cell state$c_{t}$. This mechanism not only ensures effective capture of long-term dependencies but also contributes significantly to training stability.


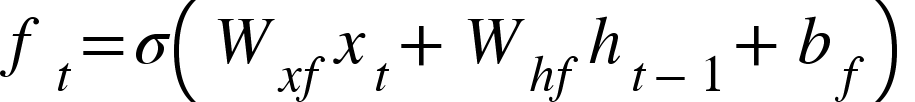


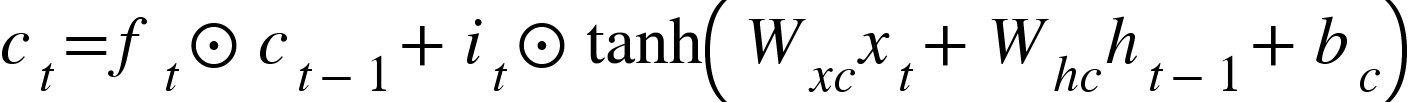


The output gate governs the influence of the cell state $c_{t}$ on the hidden state $h_{t}$, ensuring that only the most pertinent information is released at time $t$. Its formulation is given in Equation from above, and the final hidden state $h_{t}$is computed as follows:


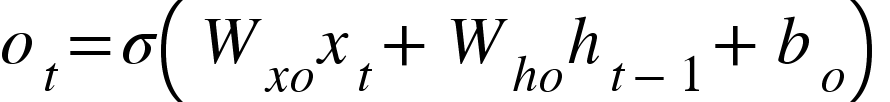


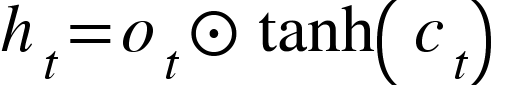


Convolutional neural networks (CNNs) have demonstrated exceptional efficacy in data processing tasks. Their architecture is chiefly composed of three core components: the input layer, the hidden layers and the output layer. The input layer serves to receive and pre-process incoming data, ensuring that it is appropriately normalised and thereby establishing a robust foundation for subsequent convolutional operations. The hidden layers form a composite structure comprising multiple functionally distinct modules—namely convolutional layers, pooling layers and fully connected layers—arranged in an alternating, hierarchical fashion. In practice, a convolutional layer is typically followed by a pooling layer, which in turn is succeeded by another convolutional layer, and so forth, thereby constructing a deep network capable of progressively extracting increasingly abstract features.

Within each convolutional layer, individual neurons maintain localised receptive fields over the input data. Each neuron computes a weighted sum of these local inputs—using learned filter coefficients—and adds a bias term, an operation that mirrors the mathematical convolution operator and which gives the network its name. The output layer then transforms the feature representations produced by the hidden layers into the final predictions or classifications required by the task at hand.

By exploiting both weight sharing and local connectivity, CNNs effect a highly efficient transformation of raw input data into a compact set of discriminative features. This not only markedly reduces the total number of trainable parameters but also accelerates the computation, thereby enabling CNNs to maintain high levels of accuracy and throughput when confronted with complex datasets. Supplementary Figure 4 illustrates the prototypical structure of a convolutional neural network.


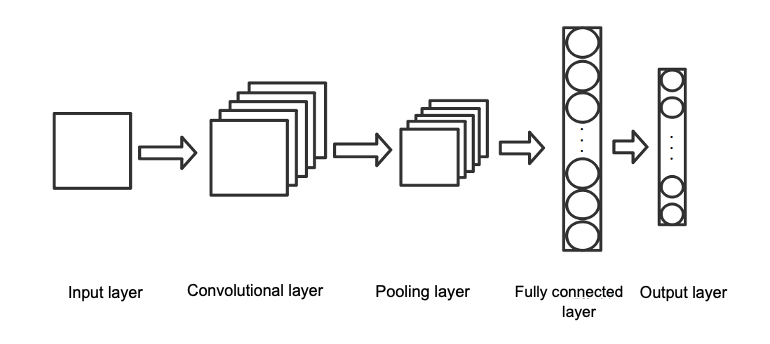


Supplementary Figure 4. | Typical convolutional neural network (CNN) structure.

In the network illustrated, each convolutional layer is immediately succeeded by a pooling layer. We denote the feature map at the $i-th$ layer by $H_{i}$ (with $H_{0}$representing the initial input). The transformation performed to obtain $H_{i}$ may be expressed as:


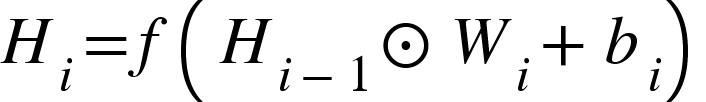


Here, $W_{i}$ is the weight vector of the $i-th$ convolutional kernel; the operator $\odot$denotes convolution between the kernel and the preceding layer’s feature map; $b_{i}$is the bias vector; and $f\left( x \right)$is the activation function applied element-wise to yield the output feature map $H_{i}$.

The pooling layer that follows executes a down-sampling operation according to either a maximum- or average-pooling strategy. In addition to effectively reducing the spatial dimensions of the feature maps—thereby lightening the computational load—pooling contributes to feature extraction by emphasising salient patterns. Crucially, it introduces local invariance, which enhances the model’s robustness to small perturbations in the input and helps mitigate overfitting by granting a measure of translational invariance. The subsampling operation may be compactly written as:


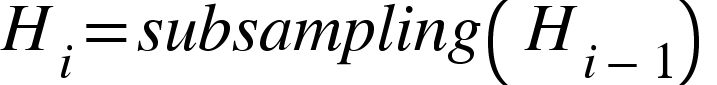


Once the cascade of convolution and pooling layers has been completed, the resulting feature representations are passed to the fully connected section of the network. This stage performs the final classification (or regression) and outputs a probability distribution $Y\left( i \right)$. Mathematically, this can be framed as a mapping


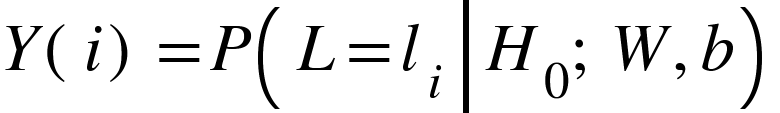


which encapsulates the progressive data transformations and feature-space compressions that project the original high-dimensional input into an optimised low-dimensional representation, preserving essential hierarchical information.

During training, the model minimises a loss function $L\left( W,b \right)$that quantifies the discrepancy between the network’s outputs and their corresponding targets. Widely used choices for this loss function include the mean absolute percentage error (MAPE) and the root mean square error (RMSE).
